# The influence of osteoporosis on mechanical complications in lumbar fusion surgery: a systematic review

**DOI:** 10.1016/j.xnsj.2024.100327

**Published:** 2024-05-03

**Authors:** Anna Filley, Avionna Baldwin, Alma Rechav Ben-Natan, Keith Hansen, Ayush Arora, Angel Xiao, Deana Hammond, Caressa Chen, Isobel Tweedt, James Rohde, Thomas Link, Sigurd Berven, Aenor Sawyer

**Affiliations:** a Department of Orthopaedic Surgery, University of California, 435 Warren Drive, Apt 11, San Francisco, CA, USA; b Department of Neurological Surgery, University of California, San Francisco, CA, USA; c Department of General Surgery, University of California, San Francisco, CA, USA; d Loyola University Medical Center; Maywood IL, USA; e Western University of Health Sciences College of Osteopathic Medicine of the Pacific, USA; f Department of Integrative Biology, University of California Berkeley, USA; g Department of Radiology and Biomedical Imagery, University of California, San Francisco, CA, USA

**Keywords:** Osteoporosis, Bone mineral density, Lumbar fusion, Instrumentation failure, Degenerative spine disease, Hounsfield units, Opportunistic screening, Spine surgery, Complications

## Abstract

**Background:**

Adults undergoing spine surgery often have underlying osteoporosis, which may be a risk factor for postoperative complications. Although these associations have been described, osteoporosis remains profoundly underdiagnosed and undertreated in the spine surgery population. A thorough, comprehensive systematic review summarizing the relationships between bone mineral density (BMD) and specific complications of lumbar fusion surgery could be a valuable resource for raising awareness and supporting clinical practice changes.

**Methods:**

PubMed, Embase, and Web of Science databases were searched for original clinical research articles reporting on BMD, or surrogate measure, as a predictor of complications in adults undergoing elective lumbar fusion for degenerative disease or deformity. Endpoints included cage subsidence, screw loosening, pseudarthrosis, vertebral fracture, junctional complications, and reoperation.

**Results:**

A total of 71 studies comprising 12,278 patients were included. Overall, considerable heterogeneity in study populations, methods of bone health assessment, and definition and evaluation of clinical endpoints precluded meta-analysis. Nevertheless, low BMD was associated with higher rates of implant failures like cage subsidence and screw loosening, which were often diagnosed with concomitant pseudarthrosis. Osteoporosis was also a significant risk factor for proximal junctional kyphosis, particularly due to fracture. Many studies found surgical site-specific BMD to best predict focal complications. Functional outcomes were inconsistently addressed.

**Conclusions:**

Our findings suggest osteoporosis is a significant risk factor for mechanical complications of lumbar fusion. These results emphasize the importance of preoperative osteoporosis screening, which allows for medical and surgical optimization of high-risk patients. This review also highlights current practical challenges facing bone health evaluation in patients undergoing elective surgery. Future prospective studies using standardized methods are necessary to strengthen existing evidence, identify optimal predictive thresholds, and establish specialty-specific practice guidelines. In the meantime, an awareness of the surgical implications of osteoporosis and utility of preoperative screening can provide for more informed, effective patient care.

## Highlights

• Patients with degenerative spinal disease often have underlying osteoporosis.
• Osteoporosis is an important risk factor for mechanical complications after lumbar fusion.
• Routine preoperative screening allows interventions that can reduce complication risk.
• Alternative screening recommendations may be necessary for surgical spine patients.
• Standardized methods of bone health evaluation and complication reporting are needed.

## Background

Osteoporosis is a highly prevalent, age-related skeletal disorder characterized by a progressive loss of bone mass and increased susceptibility to fractures [1]. As the global population ages, the prevalence and significance of osteoporosis and other age-related degenerative conditions will continue to rise [1,2]. This demographic shift is of particular significance to the spine surgeon, who will be increasingly faced with the challenge of treating patients with degenerative spinal pathologies, poor bone quality, and sequelae of osteoporosis. These conditions are frequently comorbid, with one recent systematic review estimating 79% of spine surgery patients over the age of 50 have osteoporosis or low bone mass [3]. Previous studies have reported higher rates of complications, longer hospitalizations, more frequent readmissions and reoperations, and increased total healthcare costs in osteoporotic patients following spine surgery [[4], [5], [6]]. Osteoporosis has also been suggested to be an independent risk factor for mechanical complications like cage subsidence (CS), pedicle screw loosening (SL), pseudarthrosis, vertebral compression fracture (VCF), and proximal junctional kyphosis (PJK) or failure (PJF) [[7], [8], [9], [10], [11]].

However, despite this prevalence and association with poor outcomes, osteoporosis remains profoundly underdiagnosed and undertreated in the spine surgery population [12,13]. Although many surgeons anecdotally recognize the challenges of instrumenting osteoporotic bone and may modify their surgical plan in the setting of this diagnosis, dedicated bone health assessments are infrequently performed preoperatively [[14], [15], [16]]. Underutilization of osteoporosis screening may be related to a variety of issues including logistical difficulties, concerns about the accuracy of lumbar T-scores in the degenerative spine, or lack of consensus regarding the implications of low bone density for surgical management. In the absence of clear specialty-specific guidelines addressing bone density in elective lumbar fusion, many surgeons may feel uncomfortable assuming the responsibility for osteoporosis screening and treatment [17,18]. Inadequate insurance coverage and reimbursement practices can also discourage providers from ordering diagnostic testing or prescribing pharmacologic therapies, and may make patient adherence to treatment cost-prohibitive [19,20].

Consequently, while an association between osteoporosis and surgical complications may seem intuitive, it is not reflected in current practices, specialty guidelines, or healthcare policies. Addressing this gap will require engagement of patients, surgeons, and policymakers regarding the importance of bone health in spine surgery and the utility of preoperative screening for preventing complications. The purpose of this manuscript is therefore to summarize existing literature on osteoporosis in lumbar fusion, focusing on mechanical complications that can be attributed to poor bone health. Rather than concentrating on a single outcome in isolation, the authors felt a comprehensive review that encompasses the spectrum of osteoporosis-related complications is necessary to put the significance of this condition into perspective and advocate for changes in the standard of care. Moving forward, these findings can serve as a reference to inform current practices, identify areas in need of further study, and ultimately provide for more consistent, effective, and evidence-based patient care.

## Methods

### Literature search strategy

PubMed, Embase, and Web of Science databases were searched for original research articles reporting on the risk of specific mechanical complications of lumbar fusion surgery in relation to bone mineral density (BMD), or surrogate measurement. Details on individual search strategies are provided in Supplementary File, Table S1. Articles were independently screened for eligibility by 2 reviewers (A.F. + A.B.) using the criteria in Table 1.

**Table 1:** Details of article selection criteria

Inclusion criteria
1) Original research articles with a minimum study size of 10 patients
2) Study population must be adult patients undergoing elective instrumented lumbar fusion surgery (includes standalone interbody fusions)
3) Measured primary outcome(s) of the incidence of specific radiographic surgical complication(s) or need for revision surgery
4) Comparison between osteoporosis or low bone mass group and normal bone density group* or risk stratification based on bone density (or surrogate measure)
*Studies must clearly specify the diagnostic criteria for osteoporosis (ex., T-score ≤ -2.5 or history of fragility fracture)
5) Publication date from 2002 onward

Exclusion criteria

1) Reviews, case reports, biomechanical studies, cadaveric research
2) Surgical interventions including non-instrumented procedures (ex., decompression alone, vertebroplasty) or those performed for indications other than degenerative disease (ex., infection, trauma, malignancy)
3) Investigations of only osteoporotic patients (no internal control group)
4) Failure to specify the incidence of specific complications (i.e., reporting generalized results for “all complications”)
5) Studies reporting on mixed populations without stratifying results based on osteoporosis assessment
6) Studies that did not perform a baseline evaluation of bone health for all eligible participants

### Data extraction and outcome measures

Data was extracted by the first author (A.F.) and validated by 2 additional authors (K.H. + J.R.). Variables included information related to (1) study design and setting; (2) patient demographics; (3) treatment characteristics: surgical procedures and perioperative anti-osteoporosis therapies; (4) prognostic factor assessment: imaging modality, anatomical site(s), and cutoff thresholds or diagnostic criteria used (if applicable); and (5) primary outcomes evaluated: imaging modality, timing, and diagnostic criteria. Mechanical complications reported by at least 2 studies, with relevant statistics, were considered for analysis (Table 2). Missing data were sought out through contact with corresponding authors.

**Table 2:** Primary outcomes included

Interbody cage subsidence Screw loosening Pseudarthrosis New vertebral fracture Junctional pathologies (adjacent segment disease, proximal junctional kyphosis or failure)
Revision surgery

For each primary outcome we presented studies’ findings of prognostic effect, including estimated odds ratio (OR) for binary outcomes and mean differences or unit odds ratio (UOR) for continuous outcomes. Event rates for dichotomous data were used to generate forest plots for each outcome. Overall, data were summarized but not pooled due to substantial variability in study methodologies.

### Methodological quality assessment

Evidence quality was evaluated using the system proposed by the Grading of Recommendations Assessment, Development and Evaluation (GRADE) working group [21]. Using major GRADE criteria, articles were evaluated in the context of methodological domains thought to be highly important for studies of prognostic factors, including patient selection and comparability of subjects, prognostic factor assessment, appropriateness of clinical endpoints, data collection and analysis practices, and disclosure of funding [[22], [23], [24]]. Assessments were performed independently by 2 reviewers (A.F. + A.R.), with discrepancies reconciled in discussion.

## Results

### Study selection

**Fig. 1:**
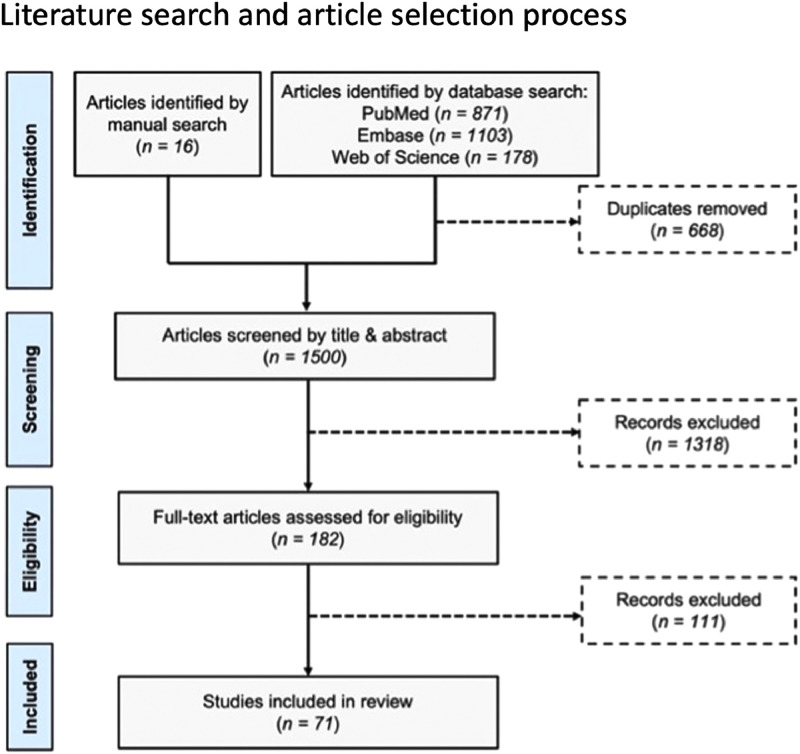
Flow diagram depicting literature search and article selection process.

**Fig. 2:**
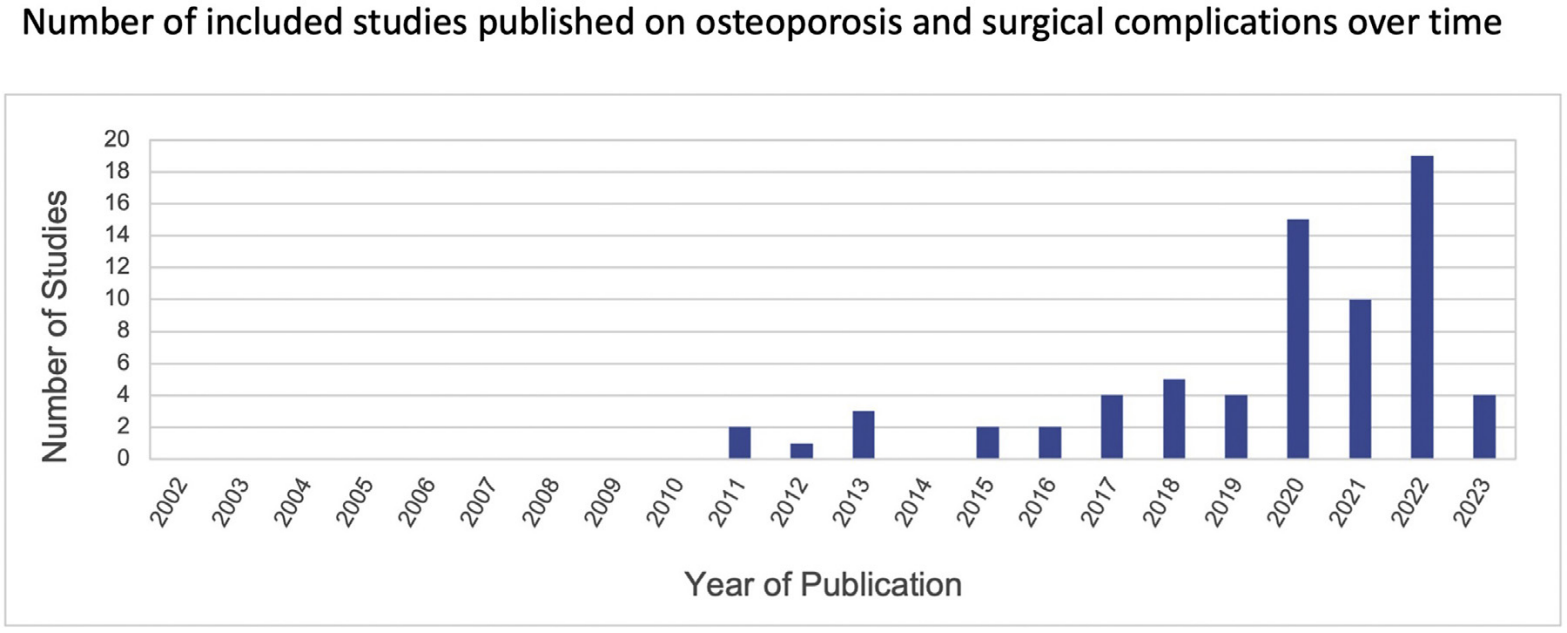
Number of included studies published on osteoporosis and surgical complications over time *Among 71 total studies. The electronic search included the years 2002 to 2023.*

The article selection process is detailed in Figure 1. An initial database search returned 2,112 citations, 1,500 after removal of duplicates. An additional 16 articles were identified manually. Screening by title and abstract left 182 references for full-text review. Ultimately, 71 studies satisfied our inclusion and exclusion criteria. Notably, 48 of these (67.6%) were published since the year 2020 and nearly one-third since 2022 (Figure 2).

### Study characteristics and quality assessment

Main characteristics of the included studies are presented in Table 3. In total, 12,278 patients (63% female) were included, with mean ages ranging between 44.9 and 72.5 years. All patients underwent primary or revision lumbar fusion for degenerative disease or deformity.

**Table 3:** Characteristics of included studies

Author	Year	Study Design	No. patients	Age, years *Mean ± SD (range)*	Bone Health Assessment		Osteoporosis treatment	Surgical Intervention	Clinical Follow-up		Primary Outcomes	Radiographic assessment
					Diagnostic modality	Measurement location			*Mean ± SD (range)*	Min		
Alan [31]	2022	Retrospective review	55	63.6 ± 10.1 (18+)	CT (HU) DXA	LIF segment NR	3.6% (2/55)	Single or multilevel LLIF	13.3 ± 8.5 mo	1 y	Cage subsidence	X-ray *±* CT
Amorim-Barbosa [26]	2022	Retrospective review	165	*CS: 52 ± 16, No CS: 49 ± 12*	CT (HU) DXA	Global lumbar Lumbar	NR	Single or multilevel TLIF/PLIF	NR	6 mo	Cage subsidence	X-ray
Barton [118]	2017	Retrospective review	94*	58.6 ± 12.7 (23-82)	DXA or ultrasound	NR	NR	Multilevel posterior or AP fusion with osteotomy	30 mo	2 y	Junctional disease	NR
Bokov [119]	2018	Retrospective review	250	52 ± 12.1 (28-74)	CT (HU)	L3	NR	Short-segment lumbar fusion ± LIF	NR	1.5 y	Screw loosening	CT
Chen [60]	2011	Retrospective review	109	53.4 (28-72)	DXA	Lumbar	NR	L4/5 PLIF	39.3 mo (24-52 mo)	2 y	Junctional disease	X-ray
Chen [37]	2023	Retrospective review	174	63.5 ± 7.8 (50+)	MRI (VBQ) DXA	Global lumbar Lowest T-score	NR	Short-segment lumbar fusion with PLIF	14.6 mo (12-37 mo)	1 y	Screw Loosening	X-ray
Cho [34]	2018	Retrospective review	86	*Osteoporosis: 66.1 ± 8.0, Normal BMD: 65.8 ± 7.8*	DXA	Lumbar	19.7% (17/86)	Single-level PLIF	NR	2 y	Cage Subsidence Screw loosening Pseudarthrosis	CT X-ray or CT X-ray and CT
Choi [44]	2023	Retrospective review	79	*Osteoporosis: 69.9 ± 6.9, Low BMD: 62.6 ± 7.8, Normal BMD: 56.6 ± 7.7*	CT (HU) Partial (38%) DXA	L1 NR	44.3% (38/79)	Single-level TLIF	40.3 mo	2 y	Pseudarthrosis	CT
Duan [53]	2020	Retrospective review	54	64.9 ± 7.6 (50+)	CT (HU)	UIV, UIV+1, UIV+2	NR	Long posterior fusion	*3.19 ± 1.14 y*	2 y	Junctional disease	X-ray
Ehresman [66]	2020	Case-control	90	*Case: 63.5 ± 8.2, Control: 63.1 ± 10.6 (18+)*	MRI (VBQ) Partial (39%) DXA	Global lumbar FN	NR	Multilevel lumbar fusion	*Case: 6.1 ± 4.1 y, Control: 3.5 ± 1.1 y*	2 y	Reoperation	N/A
Guha [27]	2022	Retrospective review	89	61.6 ± 10.5 (18+)	CT (HU) DXA	LIF segment FN	NR	Single or multilevel LLIF	19.9 ± 13.9 mo	6 mo	Cage subsidence Reoperation	X-ray
Ha [45]	2019	Retrospective review	157	68.0 ± 6.3 (60+)	DXA	Lowest T-score	NR	Long posterior fusion	53.2 ± 34.3 mo (24–152)	2 y	Junctional disease	X-ray
Hiyama [120]	2022	Retrospective review	59	68.9 ± 10.6 (25-89)	CT (HU)	LIF segment (EP)	NR	Single-level LLIF	NR	1 y	Cage subsidence	X-ray and CT
Hiyama [54]	2022	Retrospective review	52	70.2 ± 9.2 (20+)	CT (HU)	UIV, UIV+1, UIV+2	NR	Staged multilevel LLIF and long posterior fusion	17.7 ± 9.5 mo	1 y	Junctional disease	X-ray
Hu ^39(p)^	2022	Retrospective review	242	60.5 ± 13.3 (18+)	MRI (VBQ) Partial (21%) DXA	Global lumbar TH, FN, lumbar	6.6% (16/242)	Single-level TLIF	35.77 ± 16.33 mo	2 y	Cage subsidence	X-ray
Hyun [121]	2016	Retrospective review	44	*PJK: 64.7 ± 7.3, No PJK: 63.4 ± 7.3 (20+)*	DXA	NR	NR	Long posterior or AP fusion	NR	2 y	Junctional disease	X-ray
Jones [122]	2021	Retrospective review	347	61.7 ± 11.1 (18+)	QCT (vBMD)	Mean L1/2; LIF segment (EP)	NR	Single or multilevel LLIF	NR	5 mo	Cage subsidence	X-ray or CT
Jones [123]	2022	Retrospective review	89	65.94 ± 10.44 (18+)	MRI (VBQ, EBQ) QCT (vBMD)	Global lumbar; LIF segment (EP) Mean L1/2	NR	Single or multilevel standalone LLIF	NR	5 mo	Cage subsidence	X-ray or CT
Jung [32]	2019	Retrospective review	84	*Osteopenia: 65.3 ± 7.2, Normal BMD: 64.2 ± 10.2*	DXA	FN	NR	Single-level D-LIF	*Osteopenia: 44.3 ± 14.3 mo, Normal BMD: 43.2 ± 12.2 mo*	2 y	Cage subsidence Pseudarthrosis	X-ray X-ray and CT
Kim, MC [124]	2013	Retrospective review	104	61.3 ± 9.8 (38-79)	DXA	NR	NR	1 or 2-level MI-TLIF	31.3 ±10.8 mo (24-45 mo)	2 y	Cage subsidence	X-ray
Kim, HJ [33]	2013	Retrospective review	364	*PJK: 53.3 ± 14.5, No PJK: 48.9 ± 15.0 (18+)*	DXA	NR	NR	Long posterior or AP fusion	3.5 y (2-6 y)	2 y	Junctional disease	X-ray
Kim, DK [125]	2017	Retrospective review	49	*PJK: 62.5 (56-69), No PJK: 61.9 (54-69)*	DXA	NR	NR	Long posterior or AP fusion	*PJK: 47.7 ± 23.4 mo, No PJK: 45.6 ± 25.6 mo*	2 y	Junctional disease	X-ray
Kim, KH [38]	2022	Retrospective review	113	65.2 ± 10.8	CT (HU) Partial (73%) DXA	Global lumbar Lumbar	NR	Single or multilevel lumbosacral fusion	NR	6 mo	Screw Loosening	X-ray
Kotheeranurak [126]	2021	Retrospective review	107	67.4	DXA	NR	NR	Single or multilevel OLIF	34.2 mo (24–72 mo)	2 y	Cage subsidence	X-ray and CT
Kuo[59]	2023	Retrospective review	116	64.1 ± 6.8 (50+)	MRI (VBQ) Partial (61%) DXA	Global lumbar Lumbar, TH, FN	NR	Thoracolumbar fusion	*PJK/PJF: 27.6 ± 15.4 mo, No PJK/PJF: 24.7 ± 12.0 mo*	1 y	Junctional disease	X-ray
Kurra [47]	2022	Retrospective review	92	64 (42-81)	CT (HU) Partial (52%) DXA	UIV-1, UIV, UIV+1 NR	NR	Long posterior fusion	1.5 y (0.2-4 y)	NR	New VCF Junctional disease	CT X-ray
Lee [127]	2020	Retrospective review	59	69.6 ± 5.9 (60+)	DXA	NR	NR	Long posterior fusion	87.4 ± 37.5 mo	2 y	Pseudarthrosis	CT
Li [42]	2023	Retrospective review	56	56.6 ± 11.96	CT (HU)	LIF segment, screw insertion point	NR	L4/5 OLIF	12.2 mo (11-13.5 mo)	1 y	Screw loosening	CT
Liu [84]	2020	Prospective cohort	105	58.5 (43-71)	Micro CT (BS/TV) DXA (BMD)	Spinous process specimen (ex-vivo) Lumbar, FN	NR	Single-level PLIF	NR	2 y	Pseudarthrosis	CT
Löffler [128]	2021	Case-control	46	69.9 ± 9.1 (48-85)	CT (vBMD)	Global, segmental (L1-4)	NR	Short-segment lumbar fusion	Median 365 d (71-2225 d)	6 mo	Screw loosening	X-ray or CT
Luo [46]	2020	Retrospective review	669	59.92 ± 7.41	DXA	Lumbar	NR	Short-segment fusion with PLIF	2.7 ± 1.1 y (2–4 y)	2 y	New VCF	X-ray
Matsukawa [39]	2018	Retrospective review	92	63.4 ± 14.8 (31-88)	CT (HU sum x1000) DXA	Screw trajectory Lumbar, FN	NR	Single-level PLIF, pedicle screw fixation using CBT	25.6 ± 10.2 mo	1 y	Screw loosening	CT
Meredith [48]	2013	Case-control	40	*Case: 66 (49-88), Control: 62 (49-80)*	CT (HU)	Global thoracolumbar, fracture level	17.5% (7/40)	Multilevel posterior or AP fusion	NR	6 mo	New VCF	X-ray, CT, MRI, or bone scan
Mi [129]	2017	Case-control	36	*Case: 53 (23-73), Control: 54 (25-71)*	CT (HU)	Global lumbar, LIF segment	0	Single-level TLIF with unilateral fixation	NR	6 mo	Cage subsidence	CT
Mikula [55]	2021	Retrospective review	150	66 ± 7.4 (50+)	CT (HU) Partial (55%) DXA	Mean L3/4, UIV/UIV+1 FN, TH, lumbar	55% (83/150)	Long instrumented fusion	31.8 ± 20.2 mo	1 y	Junctional disease	X-ray
Mikula [56]	2022	Retrospective review	81	66 ± 6.9 (50+)	CT (HU) Partial (70%) DXA	Mean L3/4, UIV/UIV+1 FN, TH, lumbar	56% (45/81) pre-op 22.2% (18/81) post-op	Long instrumented fusion	38 ± 25 mo	1 y	Junctional disease	X-ray
Mugge [64]	2022	Retrospective review	532	*Osteoporosis: 69 ± 11, No osteoporosis: 59 ± 19 (18+)*	DXA	Femoral head	27% (144/532)	Long thoracolumbar fusion	18.5 ± 68.7 mo	NR	Reoperation	N/A
Nguyen [130]	2015	Case-control	20	Case: 44.4 ± 12.14, Control: 45.4 ± 10.65	CT (HU)	Global lumbar (L1-3), UIV/UIV-1	NR	L4-S1 posterolateral fusion	NR	1 y	Pseudarthrosis	Intraoperative or radiographic
Oh [131]	2015	Retrospective review	102	65.17 ± 8.59 (37-86)	DXA	Lumbar (LIF segment)	NR	Single or multilevel PLIF	4.1 y (1.4-7.7 y)	1 y	Cage subsidence	CT
Okano [132]	2020	Retrospective review	96	Median 68 [IQR 62.2-74.3]	QCT (vBMD)	Mean L1/2, LIF segment (Tb, EP)	NR	Single or multilevel standalone LLIF	Median 26 mo [IQR 8-102 mo]	6 mo	Cage subsidence	X-ray or CT
Otsuki [133]	2021	Retrospective review	85	*Nonunion: 72.1 ± 6.9, Union: 68.2 ± 8.4 (50+)*	CT (HU)	LIF segment	NR	L4/5 TLIF	NR	1 y	Pseudarthrosis	X-ray and CT
Park, MK [66]	2019	Prospective cohort	784	63.3 (20-85)	DXA†	Lumbar (LIF segment)	NR	Single or multilevel TLIF	NR	1.5 y	Cage subsidence	X-ray and CT
Park^81^, SJ [52]	2020	Retrospective review	63	67.2 ± 6.3 (50+)	DXA	NR	NR	Long posterior or AP fusion	51.7 ± 33.1 mo	2 y	Junctional disease	X-ray
Pisano [134]	2020	Retrospective review	89	59.9 (50+)	CT (HU)	L1	NR	Single or multilevel TLIF	27 mo	1 y	Cage subsidence	CT
Pu [30]	2022	Retrospective review	71	59.6 ± 10.1	CT (HU) DXA	Global lumbar, LIF segment Lumbar, forearm	NR	L4/5 PLIF	13.6 ± 5.1 mo	1y	Cage subsidence	CT
Ran [135]	2022	Retrospective review	70	59 ± 10.4	CT (HU)	Global lumbar, LIF segment (Tb, EP)	8.57% (6/70)	L4/5 OLIF	15.4 ± 6 mo (12-40)	1 y	Cage subsidence	CT
Rentenberger [65]	2020	Retrospective review	133	*Revision: 68.6 ± 10.6, No revision: 66.3 ± 10.6 (18+)*	QCT (vBMD)	Mean L1/2	NR	Single or multilevel standalone LLIF	NR	1 y	Cage subsidence Reoperation	X-ray or CT
Sakai [40]	2018	Retrospective review	52	68.2 ± 10.1 (44-83)	CT (HU) DXA	Screw trajectory Lumbar	17.3% (9/52)	Single-level PLIF	NR	3 mo	Screw loosening	CT
Salzmann [136]	2019	Case-control	63	*Case: 66.4 ± 8.5, Control: 65.3 ± 7.9*	QCT (vBMD)	Mean L1/2, S1, sacral ala	NR	Multilevel posterior fusion to S1	NR	6 mo	New VCF	NR
Shin [137]	2022	Retrospective review	478	65.0 ± 10.6 (22-88)	CT (HU)	L4	NR	Short-segment lumbar fusion with PLIF	43.2 ± 27.25 mo (12-113)	1 y	Screw loosening	X-ray and CT
Wang, H [57]	2016	Retrospective review	98	*PJK: 62.3 ± 6.8* *No PJK: 62.5 ± 7.5 (50+)*	DXA	NR	NR	Long posterior fusion	2.8 y (2-6)	2 y	Junctional disease	X-ray
Wang, H [61]	2017	Retrospective review	237	53.2 ± 10.8 (37-69)	DXA	NR	NR	1 or 2-level TLIF or PLIF	*Adjacent disease: 2.6 ± 0.2 y, No adjacent disease: 2.5 ± 0.3 y*	2 y	Junctional disease	X-ray
Wang, Q [138]	2020	Retrospective review	104	63.2 (49-80)	CT (HU)	L1	0% (0/104)	Long instrumented fusion	35.7 mo	2 y	Junctional disease	X-ray
Wang, SK [63]	2022	Retrospective review	821	*Early revision: 68.1 ± 11.6, Late revision: 66.9 ± 9.5, No revision: 64.9 ± 11.1 (18+)*	DXA	NR	4.5% (37/821)	Short-segment lumbar fusion with TLIF	NR	2 y	Reoperation	
Xi [139]	2020	Retrospective review	68	61.1 ± 13.3 (18+)	CT (HU)	Global lumbar, LIF segment	Criteria for exclusion	Single-level LLIF	25.3 ± 10.4 mo	1 y	Cage subsidence	X-ray
Xie [28]	2022	Retrospective review	279	50.9 ± 8.8 (18+)	CT (HU) Partial (24%) DXA	Global lumbar, segmental (L1-4) FN and/or lumbar	NR	Single-level TLIF	Median 18 mo [12-40]	1 y	Cage subsidence	NR
Xu [43]	2020	Retrospective review	143	*SL: 62.0 ± 6.7, No SL: 62.0 ± 6.4 (50+)*	CT (HU)	L3 (vertebral body and pedicle)	NR	L3-5 posterolateral fusion	NR	1 y	Screw loosening	X-ray
Xu [35]	2022	Retrospective review	78	63 (45-80)	DXA	Lumbar, TH	NR	Long posterior fusion	NR	2 y	Screw loosening	X-ray *±* CT
Yagi, [140]	2011	Retrospective review	157	46.9 (22-81)	DXA	FN	NR	Long posterior, anterior, or AP fusion	4.3 y (2-12)	2 y	Junctional disease	X-ray
Yagi [141]	2012	Retrospective review	76	48.8 (23-75)	DXA	FN	NR	Long posterior, anterior, or AP fusion	7.3 y (5-14)	5 y	Junctional disease	X-ray
Yagi [142]	2018	Retrospective review	113	62.2 (20+)	DXA	FN	NR	Long thoracolumbar fusion	NR	2 y	Junctional disease	X-ray
Yao [143]	2020	Retrospective review	93	66.5 ± 12.2 (18+)	DXA	NR	NR	1-2 level MI-TLIF	36.9 ± 5.7 mo (24-46)	2 y	Cage subsidence	X-ray
Yao [49]	2021	Retrospective review	63	58.4 ± 14.9 (18+)	CT (HU)	Mean UIV/UIV+1	NR	Long posterior fusion	13.1 mo	1 y	Junctional disease	X-ray
Ye [62]	2021	Case-control	1258	56.4 ± 12.4 (20-87)	DXA	NR	NR	TLIF	35.0 ± 17.8 mo (24-123)	2 y	Junctional disease	NR
Yuan [36]	2021	Retrospective review	130	62.89 ± 7.08 (40-79)	DXA	NR	NR	Long posterior fusion	34.4 mo (12-98)	1 y	Screw loosening	X-ray *±* CT
Yuan [51]	2021	Retrospective review	84	*PJK: 63.53 ± 7.33, No PJK: 62.69 ± 6.4 (40+)*	DXA	NR	NR	Long posterior fusion	40.83 mo	2 y	Junctional disease	X-ray
Zhang [58]	2022	Retrospective review	333	*PJK 74 ± 6, No PJK 70.6 ± 4.2* (65+)	CT (HU)	UIV	NR	Multilevel posterior fusion	24.2 mo (18-46)	1.5 y	Junctional disease	X-ray
Zhao [144]	2022	Retrospective review	242	*Severe CS: 69.1 ± 9.9, Mild CS: 66.3 ± 10.7, No CS: 64.5 ± 9.1*	DXA	Lowest (hip)	NR	L4/5 OLIF	NR	1 y	Cage subsidence	X-ray
Zhou [29]	2021	Retrospective review	76	56.1 ± 10.4 (29-81)	CT (HU) DXA	Global lumbar, L1, LIF segment Lowest T-score	NR	Single or multilevel standalone OLIF	28.2m ± 9.3m	6m	Cage subsidence	X-ray ± CT
Zou [145]	2020	Retrospective review	503	61.2 ± 6.7 (50–83)	CT (HU)	Global and segmental (L1-4)	NR	Short-segment lumbar fusion ± PLIF	NR	1y	Screw loosening	X-ray
Zou [41]	2020	Retrospective review	252	62.4 ± 6.7 (50-83)	CT (HU) DXA	Global lumbar Lumbar, lowest T-score	NR	Short-segment lumbar fusion ± PLIF	NR	1y	Screw loosening	X-ray

Data presented describe entire study population unless otherwise specified as cohort statistics. Surgery types include long (5+ levels) posterior, anterior, or combined (AP) fusion, short-segment lumbar fusion, and lumbar interbody fusion (LIF); all LIF performed with supplemental screw fixation unless indicated to be a standalone procedure. LIF types include: anterior lumbar interbody fusion (ALIF), lateral lumbar interbody fusion (LLIF), direct lateral interbody fusion (D-LIF), oblique lateral interbody fusion (OLIF), posterior lumbar interbody fusion (PLIF), transforaminal lumbar interbody fusion (TLIF), and minimally invasive TLIF (MI-TLIF).

Abbreviations: not recorded (NR), dual-energy x-ray absorptiometry (DXA), computed tomography (CT) quantitative CT (QCT), Hounsfield Units (HU), bone mineral density (BMD), volumetric BMD (vBMD), magnetic resonance imaging (MRI), vertebral bone quality (VBQ), endplate bone quality (EBQ), endplate (EP), trabecular (Tb), femoral neck (FN), total hip (TH), upper instrumented vertebra (UIV), cage subsidence (CS), screw loosening (SL), vertebral compression fracture (VCF), proximal junctional kyphosis (PJK), proximal junctional failure (PJF), inter-quartile range (IQR)

⁎ Ninety-four operations in 88 patients

† Preoperative DXA obtained in 84.3% (661/784) of study patients according to criteria: 1) all age 60 or older (n=598) or 2) age younger than 60 with comorbidity or chronic medication with potential to cause osteoporosis (n=63)

In terms of prognostic factor assessment, 38 studies used BMD measured by dual-energy X-ray absorptiometry (DXA), the current gold-standard for diagnosing osteoporosis [25]. Only 22 studies specified the anatomic site(s) of DXA scans, typically either the proximal femur or lumbar spine for all participants. Five studies used each patient's lowest T-score, noting severe degeneration, scoliosis, or instrumentation to variably preclude certain measurements. Citing concerns regarding the availability or accuracy of DXA, 42 studies investigated alternative techniques, most commonly opportunistic measurement of Hounsfield Units (HU) from preoperative CT scans (Table 4).

**Table 4:** Overview of methods used for bone health assessment, and their proportions

Tool	Metric *measured variable (units)*	Measurement location *Including standardized protocol and experimental sites*	Diagnostic criteria *Thresholds for identifying poor bone health*	No. studies with complete population data
Gold-standard				
**DXA**	BMD (g/*cm*^2^) T-score	Femoral neck/total femur region, lumbar spine, distal radius*	Normal: T-score ≥ -1 Osteopenia: -2.5 < T-score < -1 Osteoporosis: T-score ≤ -2.5†	38 ‡
Alternatives investigated				
**QCT**	vBMD (mg/cm^3^)	L1-2 Experimental sites: fusion-level vertebral endplates, sacral ala	Normal: vBMD ≥ 120 mg/cm^3^ Low bone mass: 80 mg/cm^3^ < vBMD < 120 mg/cm^3^ Osteoporosis: vBMD ≤ 80 mg/cm^3^§	5
**CT**	Hus	Variable Experimental sites: thoracolumbar spine (mean global, segmental, individual levels) including intra-vertebral sites (endplates, pedicles, screw trajectory)	Normal: HU > 120; 135 Low bone mass: 90; 110 < HU < 120; 135 Osteoporosis: HU < 90; 110 ǁ	33
**MRI**	VBQ score	T1-weighted sagittal scans: median L1-4 signal standardized against CSF at L3 Experimental sites: fusion-level vertebral endplates	Research method	5
**Micro-CT**	BS/TV, BS/BV, Tb.Th, Tb.N, Tb.Sp	Spinous process specimen obtained from index surgery (ex-vivo)	Research method	1

According to International Society for Clinical Densitometry (ISCD) guidelines [146] routine BMD screening is indicated for all females over age 65 and males over age 70, as well as younger patients with risk factors for low bone mass. The current best-established standard for diagnosing osteoporosis or osteopenia relies on T-scores derived from areal BMD (g/cm^2^) measured by DXA, ideally of the femoral neck or lumbar spine. These thresholds can be applied in postmenopausal women and men over age 50. Alternatively, volumetric BMD (mg/cm^3^) can be directly measured by QCT. Of the 2 methods, central DXA is generally preferred for making therapeutic decisions and limiting radiation exposure, however, QCT may be considered superior to DXA in settings of severe degenerative disease or scoliosis [147]. A number of studies have suggested that fracture risk can also be assessed with CT attenuation in Hounsfield units (HU), which can be measured from CT scans obtained for other purposes that include the lumbar spine (opportunistic bone density measurement). In the absence of established protocols, methodologies for HU measurement varied widely and included standardized (mean or segmental) and patient-specific (ex., junctional vertebrae, screw trajectory, fusion-level endplates) sites. MRI and micro-CT are other techniques used to assess bone quality; as purely research methods, both follow standardized protocols but do not have established clinical correlates or guidelines for identifying at-risk patients. Additionally, while MRI metrics can be obtained via opportunistic measurement, micro-CT is an ex-vivo study and therefore cannot be used for screening preoperatively.

Study acronyms are explained in the first footnote to Table 3. Abbreviations: CSF, cerebrospinal fluid; BS/TV, bone surface / total volume; BS/BV, bone surface / bone volume; Tb.Th, trabecular thickness; Tb.N, trabecular number; Tb.Sp, trabecular separation.

⁎ Hip and spine measurements preferred; distal radius recommended only when hip and spine cannot be obtained.

† According to the World Health Organization (WHO), gold-standard T-score thresholds used for the diagnosis of osteoporosis and osteopenia [25]

‡ Includes one prospective observational study in which all patients over the age of 60 years (n=598), as well as all younger patients with risk factors for osteoporosis (n=63), underwent a preoperative lumbar DXA. In total, this amounted to 84.7% (661/784) of participants [81].

§ According to American College of Radiology (ACR) practice guidelines, vBMD (mean L1/2) thresholds for osteoporosis and low bone mass [147]

ǁ Although there has been no established consensus regarding HU thresholds for diagnosing osteoporosis or osteopenia, several large-scale studies utilizing L1 HU have suggested values of 90 or 110 for osteoporosis and 135 or 120 for low bone mass [148,149]. A recent systematic review of studies reporting on the correlation between lumbar HU and DXA T-scores identified 16 studies describing a cutoff for identifying osteoporosis (thresholds ranged 49.4–160), with a medium HU value of 114.8 (95% CI 90.9–138.7, p<.001). Notably, there was significant heterogeneity (*I^2^*=94.94%) among studies, including patient populations and location of HU measurement [150]. Another meta-analysis of studies evaluating the accuracy of osteoporosis diagnosis using CT HU compared to DXA suggested a threshold of 135 to diagnose osteoporosis; the authors similarly noted that their conclusions were significantly limited by study heterogeneity [151].

Evidence quality was designated as high, moderate, low, or very low based on GRADE criteria (Supplementary File, Table S2). Studies were frequently downgraded for risk of bias or indirectness in prognostic factor assessment, either by employing imaging-based selection criteria or lacking a gold-standard comparison. Insufficient accounting for confounders was another common reason for downgrading.

### Primary outcomes

An overview of findings for each primary outcome is shown in Table 5. Ultimately, a meta-analysis was not possible due to different non-comparable methods of prognostic factor assessment, variable definitions and timing of clinical endpoints, and the use of different cut point values for statistical analysis.

**Table 5:** Summary of findings

Outcome timing of radiographic follow-up	No. participants (studies)	Reported complication rates	Reported risk factors	
			Bone quality	Other independent risk factors
Cage subsidence, *Measured in millimeters or relative loss of disc height on X-ray and/or CT* Minimum follow-up: range 5 to 24 months	3,555 patients, 4,439 levels (24 studies: 22 RR, 1 PC, 1 CC)	8.25%–59% of levels*	Twenty-one studies found poor bone health associated with CS, and 3 did not.† DXA-diagnosed osteoporosis was a significant risk factor in 6 studies. Lumbar HU also predicted CS in 11 of 12 studies; outcomes were presented as an odds ratio (OR), often using a calculated optimal cutoff (ranged 104.2-135), or odds ratios per unit change (UOR) in HU. Among five studies comparing HU and T-scores, 4 found HU to best predict CS and one found nondominant forearm T-scores to be superior to both lumbar T-scores and mean HU. Four studies reported lower endplate density or quality in patients with CS. Several studies found a linear correlation between the amount of CS and either DXA T-scores (2 studies) or VBQ score (one study).	Age [126], BMI [143], paraspinal muscle atrophy [126], disc morphology [81,144], cage height [126,134,143] or shape [26,33, 39,81, 143], cage position disc overdistraction [144], intraoperative endplate injury [81,144], SA fusions [27,122], L5/S1 level [33], absence of endplate sclerosis [144]
Screw Loosening *Defined by peri-screw lucency on X-ray and/or CT* Minimum follow up: range 3 to 24 months	2,454 patients (14 studies: 13 RR, 1 CC)	13%–54.6% of patients	All studies found an association between bone health and SL. DXA-diagnosed osteoporosis was an independent risk factor in 2 studies. Bone density was most commonly assessed using CT or QCT (10 studies). Eight studies found lumbar HU to be an independent risk factor for SL; calculated cutoffs ranged 104-130,‡ though results were frequently presented using a UOR. Four studies measured regional bone density from the pedicle or screw trajectory, which was highly predictive of SL. All studies with data for comparison found DXA to be less predictive than alternatives.	Age [35], male sex [40,145], BMI [43], pedicle diameter [43], vertebral subluxation [36,119], cage type [119], bilateral facetectomy [119], laminectomy without interbody fusion [119], postoperative SVA [36,137] or TLK [36], number of fused levels [41,119,137,145], fusion to the sacrum [37,36,145]
Pseudarthrosis *Variable criteria, commonly included 1) dynamic motion, 2) lack of bridging bone, or 3) implant loosening on x-ray and/or CT*§ Minimum follow up: range 12 to 24 months	518 patients (7 studies: 5 RR, 1 PC, 1 CC)	5.95%–38.98% of patients	None of the studies utilizing DXA found an association between osteoporosis and fusion outcomes. Two of 3 studies using HU found a relationship with fusion, one of which showed that patients with osteoporosis (defined by L1 HU) had significantly longer mean times to fusion. § One study showed that bone quality of surgical specimens (assessed using ex-vivo micro CT) was a significant predictor of fusion status and functional outcomes.ǁ	Other instrumentation failures (SL [34,41,81,137,145], CS [81,120,135,144]); age [133], PEEK cages [127], lack of pelvic fixation [127], larger filling index [133]
New VCF *See footnotes*¶ Minimum follow up: range 2.4 to 24 months	1,084 patients (6 studies: 4 RR, 2 CC)	3.86%–11.95% of patients	All 5 studies evaluating proximal VCF found an association between bone density and fracture risk. Two of these were PJK subgroup analyses, in which T-scores and mean junctional HU independently predicted failure. Two other studies observed fracture patients to have lower HU both globally and at junctional or fracture levels. In the only study of sacral fractures, vBMD was not a risk factor.	Age [46], postoperative PJA [45], change in LL [46] Obesity (sacral fractures) [136]
Junctional Disease Adjacent Segment Degeneration *See footnotes*♯ Minimum follow up: 24 mo Proximal junctional kyphosis (PJK) or failure (PJF) *See footnotes*†† Minimum follow up: range 2.4 to 60 mo	1,604 patients (3 studies: 2 RR, 1 CC)	6.3%–22.01% of patients	No study found BMD to predict adjacent segment degeneration, though 2 studies did show a trend towards significance for BMD in patients with symptomatic disease.⁎⁎	BMI [61], hypertension [62], preoperative disc degeneration [61,62], superior facet violation [61]
	2,344 patients (20 studies: 20 RR)	11.5%–53.7% of patients	All studies reporting on junctional deformity found a relationship with poor bone health. Seven studies showed DXA-based osteoporosis was an independent risk factor. All studies of junctional HU reported lower values in patients with PJK (optimal cutoffs ranging 104-159), which better predicted complications compared to HU measured at non-junctional levels.‡‡ Two studies, one of VBQ score and another of mean UIV/UIV+1 HU, found a direct linear relationship between poor bone health and proximal junctional angle (PJA) measurements.	Age [52], BMI [45,57], smoking [118], PJA ≥0°[52], preop TLK [45,51] and SS [51], paraspinal muscle atrophy, [51,58,121] primary vs revision [118], type of osteotomy [118], UIV level [57,138], degree of deformity correction [121], postoperative fall [118]
Revision surgery *See footnotes*§§ Minimum follow up: range 6 to 24 months	1,665 patients (5 studies: 4 RR, 1 CC)	8.2%–22.4% of patients	Four studies found poor bone health to be a risk factor for reoperation. DXA-based osteoporosis was an independent risk factor in 2, and fusion-level HU in one other. Another case-control study found higher VBQ scores in case patients. One study did not find L1/2 vBMD, analyzed as a continuous or categorical variable, to predict revision within the first year after SA-LLIF.	Early revision: diabetes [63], foraminal stenosis [65] Late revision: multilevel (>2) fusion [63] Single-level LLIF: age, BMI, PEEK cage, SA-fusion [27]

Study populations consisted of patients undergoing primary or revision instrumented lumbar fusion (specific indications and procedures varied) for degenerative disease. All studies assessed osteoporosis, or surrogate measure of bone health, as a risk factor for specific surgical complications including cage subsidence, screw loosening, pseudarthrosis, adjacent-level fractures, junctional disease, and revision surgery.

Study acronyms are explained in the first footnote to Table 3. Additional abbreviations: RR, retrospective review; PC, prospective cohort; CC, case-control; BMI, body mass index; PA, proximal junctional angle; TLK, thoracolumbar kyphosis; SS, sacral slope; LL, lumbar lordosis; SVA, sagittal vertical axis; OR, odds ratio; UOR, unit odds ratio; PEEK, Polyetheretherketone.

Explanations:

⁎ Two studies provided complication rates in terms of number of patients rather than number of cages

† One study did not find DXA T-scores or lumbar HU to predict CS and discussed possible confounding factors including: (1) relatively low incidence of complications, (2) few patients with osteoporosis or low bone mass, all of whom were preoperatively referred for endocrinology evaluation and undergoing treatment at the time of surgery if indicated, and (3) patients with deficient BMD may be more likely to undergo supplemental pedicle screw fixation.

‡ One study calculated different cutoffs for female (153.5, AUC 0.88) and male (186.5, AUC 0.635) patients; another showed optimal HU thresholds varied based on number of levels fused (1-4) and the degree of postoperative residual deformity

§ One study showed that fusion took significantly longer in patients with osteoporosis (defined by HU<90) compared to low (HU between 90 and 120) and normal (HU>120) BMD; this study also demonstrated that fusion rates significantly varied based on the diagnostic criteria used

ǁ One study found lower trabecular number and higher trabecular separation in spinous process specimens of patients with nonunion; bone quality was also shown to correlate with patient-reported postoperative outcomes of pain and disability.

¶ No study provided a clear description of radiographic criteria used for diagnosis (ex., % loss of vertebral body height)

♯ Adjacent segment degeneration after 1 to 2 level TLIF or PLIF. Two of 3 studies utilized flexion-extension X-rays, one of which reported specific diagnostic criteria. The third study specified including all symptomatic cases requiring revision.

⁎⁎ One study presented T-scores of patients with and without adjacent segment degeneration (−1.23±0.23 vs. −1.12±0.19; p=.08), notably with very narrow SD and relatively better bone quality in both groups. The other study found higher rates of osteoporosis or severe osteoporosis in patients with progression of adjacent segment degeneration (30.7% vs. 17.5%; p=.069). Neither result reached statistical significance.

†† For outcomes of proximal junctional kyphosis (PJK), the most commonly utilized definition was that initially proposed by Glattes et al, [50] a proximal junctional angle (PJA), sagittal Cobb between the inferior endplate of the UIV and superior endplate of UIV+2, that is both >10° and at least 10° greater than the preoperative measurement. Proximal junctional failure (PJF) definitions varied more significantly and commonly included cases of PJK with additional signs of mechanical failure (fracture, spondylolisthesis, fixation failure) or any symptomatic PJK requiring revision.

‡‡ In 2 studies investigating both L3/4 and UIV/UIV+1 HU measurements, junctional values were the only independent risk factor.

§§ Surgical indications for revision were variably reported and included diagnoses related to hematoma, infection, pain or neurologic deficit, and construct failure. Follow-up timing was inconsistently reported. One study did not give a minimum follow-up time, instead providing a mean cohort follow-up of 18.5±68.7 months and mean time to reoperation of 32.2±64.1 months in osteoporotic patients and 24.2±36.6 months in those without osteoporosis

#### Cage subsidence

**Fig. 3:**
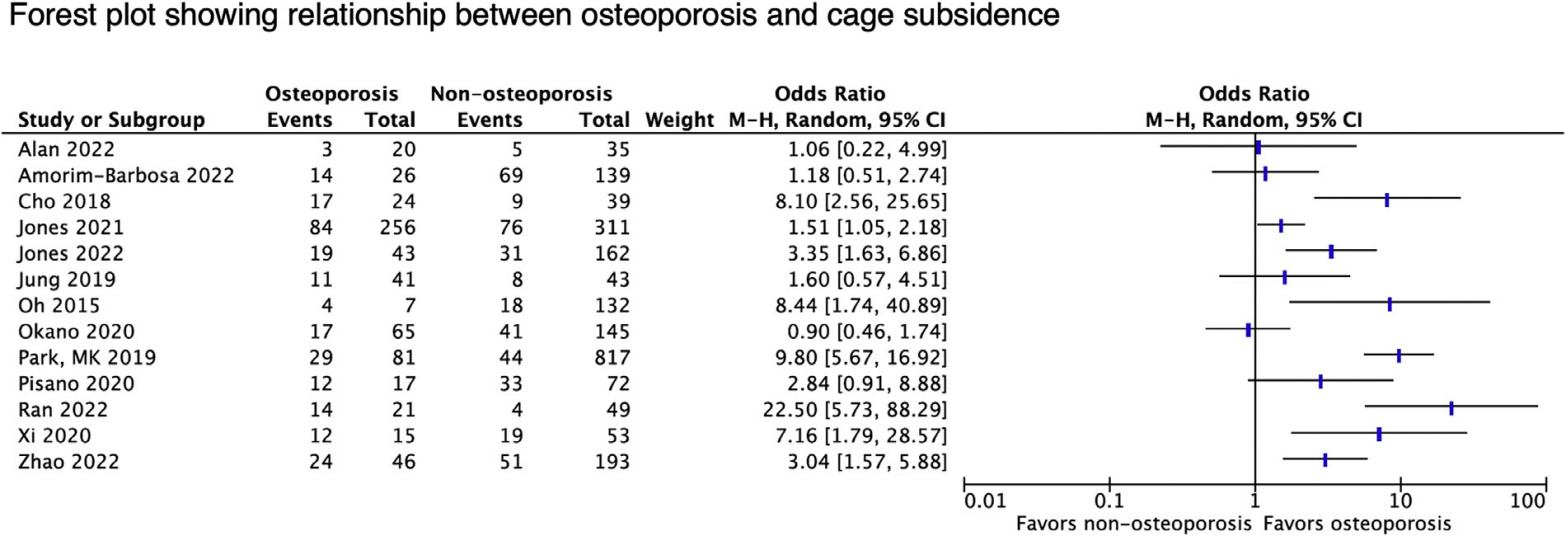
Forest plot showing relationship between osteoporosis and cage subsidence.

Cage subsidence was investigated by 24 studies in relation to BMD. Complication rates varied significantly (between 8.25% and 59% of levels), reflecting differences in surgical procedures and diagnostic criteria used (Table 6). In total, 21 studies found subsidence to be associated with poor bone health as assessed by DXA (6 studies), vBMD (4 studies), lumbar HU (10 studies), and VBQ scores (2 studies). A forest plot of all reporting relevant statistics is shown in Figure 3. Four [[26], [27], [28], [29]] of 5 studies with comparative data found HU to be more predictive of complications than traditional T-scores. Amorim-Barbosa et al. [26] showed that patients with HU<135 had a 6-fold increased risk of CS after 1-2 level PLIF or TLIF; while CS was not associated with worse functional outcomes, lower HU predicted worse disability scores and less return to work postoperatively. The remaining study, published by Pu et al. [30], found nondominant forearm T-scores to more accurately predict subsidence than mean lumbar HU (AUC 0.840 vs. 0.744), though both were independent risk factors. Notably, there were no significant differences in lumbar T-scores. Several studies investigated BMD of the fusion-level endplates as a potential predictor, reporting mixed results.

**Table 6:** Details and results of studies reporting on cage subsidence.

Study	Sample size, No. patients	Surgery type *Levels treated (No.)*	Supplemental fixation	Radiographic follow-up	Complication rates (No. segments)	Summary of results	Associated Clinical Outcomes
Alan et al. [31]	55 (97 levels)	LLIF	16 BPS, 39 SA *	6 wk, 3, 6, and 12 months	8.25% (8/97), severe (3) grade I (5), II (2), III (1) †	– Neither fusion-level HU (OR 1.01, p=.26) nor DXA-measured BMD (OR 0.81, p=.78) independently predicted CS	N/A
Amorim-Barbosa et al. [26]	165 (208 levels)	TLIF (122) or PLIF (43) *L2/3 (3), L3/4 (20), L4/5 (74), L5/S1 (68)*	Yes	NR (minimum 6 months)	50% (83/165*), 22% (36) severe	– Mean HU: 149±48 (CS group) vs 167±48 (no CS group) – HU < 135 an independent risk factor for CS (OR 6.4, p=.05)	– Lower HU associated with less return to work (p=.013) and worse ODI (p=.029) – Severe CS not associated with worse clinical outcomes
Cho et al. [34]	86	PLIF *L3/4 (13), L4/5 (60), L5/S1 (13)*	Yes	Mean time to CS: Osteoporosis: 6.3±3.4 mo Normal BMD: 6.2±3.6 mo	30% (26/86) >2mm	– Mean lumbar T-scores: -2.8±0.5 (osteoporotic cohort) vs 0.2±0.9 (normal BMD cohort), p<.001 – Higher rates of CS in osteoporotic cohort (70.8% vs 23.1%, p<.001) – Mean T-scores: -1.7±1.5 (CS group) vs. -0.4±1.4 (no CS group), p<.001	– No association between CS and VAS back (p=.703) or leg pain, ODI, or EQ-5D at final follow-up
Guha et al. [27]	89 (150 levels)	LLIF	84 BPS, 66 SA^a^	NR (minimum 6 months)	17.3% (26/89) grade: I (18), II (4), III (4)	– Lower fusion-level HU associated with CS risk in single- (UOR 0.97, p=.048), but not multilevel or SA fusions – No association between CS severity and lumbar HU (p=.91) or FN T-scores (p=.40)	N/A
Hiyama et al. [120]	59	LLIF *L2/3 (2), L3/4 (16), L4/5 (41)*	10 UPS, 49 BPS	Immediately postoperative (within 2 wk) and 1 year	33.9% (20/59) grade I 55% (11), II 25% (5), III 20% (4) - 15.3% (9/59) early, 18.6% (11/59) delayed	– Mean endplate HU: 310.2±56.5 (CS group) vs. 263.3±54.0 (no CS group), p=.004 – Higher rates of CS observed at L3/4 (50% vs. 15.4%, p=.012)	– NRS scores at 1 year significantly improved with and without CS – CS associated with lower fusion rates at 1 year (55% vs. 92.3%, p=.001)
Hu et al. [39]	242	TLIF *L1/2 (1), L2/3 (1), L3/4 (9), L4/5 (175), L5/S1 (56)*	Yes	2 wk, 3, 6, 12, and 24 mo	45.87% (111/242) grade I (102), II (6), III (3)	– Mean VBQ scores: 3.79±0.95 (CS group) vs. 2.96±0.56 (no CS group), p<.001 – Increased VBQ an independent predictor of CS (OR 14.61, p<.001) – ROC analysis: VBQ score cutoff 3.28 (AUC 0.856) best predicted CS – VBQ score moderately correlated with amount of CS (r = 0.512, p<.001)	– Severe CS associated with worse VAS back and leg pain (p<.001) but not ODI (p=.416) at 2 years
Jones et al. [122]	347 (567 levels)	LLIF *L1/2 (34), L2/3 (111), L3/4 (186), L4/5 (236)*	239 BPS, 108 SA	Between 5 and 14 mo	28.2% (160/567) grade I (124), II (24), III (12)	– Increased risk of CS with decreased fusion-level EP-vBMD (UOR 0.996, p=.032), but not Tb-vBMD (p=.163) – ROC analysis: optimal cutoff of EP-vBMD was 211.04 kg/m2 – Tb-vBMD: no significant differences (p=.163) – Standalone fusion associated with increased risk of CS (OR 2.854, p=.001)	– 1 revision surgery performed for CS
Jones et al. [123]	89 (205 levels)	LLIF *L1/2 (3), L2/3 (40), L3/4 (79), L4/5 (83)*	No	Between 5 and 14 mo	56.6% (116/205), severe 24.4% (50)	– Mean L1/2 vBMD: 97.4±34.4 (severe CS group) vs. 110.1±33.4 (no CS group), p=.021 – Mean VBQ scores: 2.67±1.08 (severe CS group) vs. 2.39±0.44 (no CS group), p=.01 – Mean EBQ scores: 5.09±2.2 (severe CS group) vs. 4.31±1.09 (no CS group), p=.001 – ROC analysis: optimal EBQ cutoff for severe CS of 5.1 (AUC 0.61) – Higher rates of severe CS in patients with EBQ > 5.1 (44.2% vs. 19.1%, p=.001) – EBQ demonstrated significant association with severe CS (OR 0.80, 95% CI 0.05-1.16, p=.037)	N/A
Jung et al. [32]	84	DLIF *L1/2 (1), L2/3 (4), L3/4 (12), L4/5 (67)*	Yes	1, 3, 6, 12, and 24 mo	22.61% (19/84), >3mm 13.0% (11)	– Mean FN T-scores: −1.7 ± 0.4 (osteopenia cohort) vs. −0.6 ± 0.6 (normal BMD cohort), p < 0.001 – No difference in osteopenia and normal BMD cohort rates of CS (26.8% vs. 18.6%, p=.439) or CS >3 mm (17.1% vs. 9.3%, p=.345), at 2 y	– No significant differences in VAS back or leg pain or ODI between cohorts at 1 and 2 y
Kim et al. [33]	104 (122 levels)	MI-TLIF *L2/3 (2), L3/4 (8), L4/5 (72), L5/S1 (40)*	Yes	Mean time to CS 7.2±8.5 mo (1–25)	32.8% (40/122): >2mm 14.8% (18), >4mm 6.6% (8)	– DXA-measured BMD was not an independent risk factor for CS >2mm (OR 0.524, p=.634)	N/A
Kotheeranurak et al. [126]	107 (137 levels)	OLIF *L2/3 (26), L3/4 (43), L4/5 (68)*	Yes	Mean time to CS 3.7±2.2 mo	41.6% (57/137)	– Mean T-scores: −0.85±0.92 (CS group) vs. −0.13±0.88 (no CS group), p=.015 – Compared to patients with T-score ≥ −1.5, increased CS risk with T-score ≤ -2.5 (OR 2.777, p=.006) but not T-scores between -1.5 and -2.5 (OR 0.429, p=.312)	– CS with less 3 month improvement in VAS back pain (p=.032); no differences in VAS or ODI at 12 mo – Fusion at 1 year in 93% (CS group) vs. 97.5% (no CS)
Mi et al. [129]	36	TLIF *L4/5 (36)*	UPS	NR (minimum 6 mo)	Case (n=18), control (n=18)	– Mean global HU: 112.4±10.08 (CS group) vs.. 140.2±10.17 (no CS group), p=.0015 – Mean fusion-level HU: 113.4±10.47 (CS group) vs. 127.9±8.13 (no CS group), p=.0075 – ROC analysis: CS best predicted by global HU 132 (AUC 0.715) and fusion-level HU 122 (AUC 0.636)	N/A
Oh et al. [131]	102 (139 levels)	PLIF *L2/3 (7), L3/4 (32), L4/5 (86), L5/S1 (14)*	Yes	1 year	>1mm 59.0% (82/139); >3mm 15.8% (22)	– Mean PLIF site BMD (g/cm2): 0.925±0.214 (CS >3mm) vs. 1.072±0.185 (CS 1-3mm, p=.049) and 1.115±0.297 (CS <1mm, p<.001) – Severely osteoporotic segments (T-score < -3.0) increased incidence of CS >3mm (OR 8.44, p=.012) – Weak negative correlation between CS and PLIF site BMD (r= -0.285, p<.001) and T-score (r = -0.252, p=.003)	– No significant correlation between CS and improvement of VAS (r = 0.017, p=.874), ODI (r = -0.006, p=.956), or SF-36 (r = 0.015, p=.886).
Okano et al. [132]	96 (210 levels)	LLIF *L1/2 (11), L2/3 (53), L3/4 (74), L4/5 (72)*	198 SA, 12 lateral plate ‡	Between 6 and 12 mo	Severe CS 27.6% (58/210)	– L1/2 vBMD: no differences in median values (p=.516) or osteoporosis category (p=.469) for patients with and without severe CS – Median fusion-level EP-vBMD (mg/cm3): 233.5 (severe CS group) vs. 257.4 (no CS group), p=.026 – Median Tb-vBMD (mg/cm3): 117.9 (severe CS group) vs. 120.5 (no severe CS group), p = 0.393 – ROC analysis: greater AUC of EP-vBMD (0.60) vs. Tb-vBMD (0.54)	N/A
Park et al. [81]	784 (881 levels)	TLIF *L1/2 (8), L2/3 (25), L3/4 (181), L4/5 (560), L5/S1 (124)*	Yes	1.5 y	CM 6.4% (56/881), CS 4.1% (36), CR 1.9% (17) §	– Osteoporosis (lumbar T-score < -2.5) an independent risk factor for CM (OR 8.73), CS (OR 5.77), and CR (OR 7.86), all p<.001 – Intra-operative endplate injury also significantly increased the risk of CS (OR 26.87, p < 0.001) and CR (OR 18.70, p < 0.001)	– 10 of 17 with CR presented with pain, 4 required revision – CS associated with fusion rates (p<.001) at 1.5 y: 97.1% no CM, 55.0 % CM, 41.7% CS, and 17.6% CR – CS associated with SL rates (p<.001) at 1.5 y: no CM 4.7%, CM 10%, CS 61.1%, CR 70.6%
Pisano et al. [134]	89	TLIF	Yes	NR (minimum 1 year)	>2mm 50.6% (45/89 a)	– Mean L1 HU: 137.71±12.83 (CS group) vs. 167.8±14.04 (no CS group), p=.002 – Mean L4 HU: 149.8 (CS group) vs. 160.8 (no CS group), p=.20 – Higher rates of CS with mean L1 HU < 110 (70.6% vs. 45.8%, p=.06), which was an independent risk factor for CS (p=.008, OR not provided).	– Fusion rates at 1 year: 82% (CS) vs. 93%, (no CS), p=.08). – No differences in persistence of radiculopathy at final follow-up: 47% (CS) vs. 38% (no CS), p = 0.383
							
							
							
Pu et al. [30]	71	PLIF *L4/5 (71)*	Yes	NR	≥2mm 23.9% (17/71)	– Mean forearm T-scores: −2.7±1.1 (CS group) vs. −1.2±1.2 (no CS group), p<.001 – Mean lumbar HUs: 96.1±45 (CS group) vs. 132.7±40.2, (no CS group) p=.015 – Mean lumbar T-scores: -1.8±1.3 (CS group) vs. -1.6±1.1 (no CS group), p=.476 – Forearm T-score (OR 0.884, p=.016) and mean HU (OR 0.752, p=.031) were independent risk factors for CS – ROC analysis: forearm T-score cutoff -2.6 (AUC 0.840), better predicted CS vs. HU cutoff 104.2 (AUC 0.744)	– No significant correlation between VAS score or improvement of JOA score at last follow-up and presence or severity of CS
Ran et al. [135]	70	OLIF *L4/5 (70)*	Yes	3 days, 3, 6, and 12 mo	>2mm 25.7% (18/70)	– Mean lumbar HU: 103.7±11.5 (CS group) vs. 142.7±30.1 (no CS group), p=.004 – No significant differences in HU of the upper (p=.314) or lower (p=.189) endplates – ROC analysis: optimal global HU cutoff for predicting CS of 113 (AUC 0.892) – CS observed in 66.6% (14/21) with mean global HU < 113 vs. 8.16% (4/49) with HU > 113	– Lower fusion rates with CS (61.1% vs. 90.4%, p=.005)
Rentenberger et al. [65]	133 (258 levels)	LLIF *T12/L1 (3), L1/2 (14), L2/3 (64), L3/4 (93), L4/5 (84)*	No	Mean time to CS 203 days (160-371)	Severe CS 26.7% (69/258)	– Mean L1/2 vBMD: 100.22±33.24 (severe CS group) vs. 108.85±33.89 (no severe CS group), p=.07	– Severe CS was not an independent risk factor for revision surgery within 1 year (OR 1.63, p=.30)
Xi et al. [139]	68	LLIF *L1/2 (2), L2/3 (9), L3/4 (26), L4/5 (31)*	Yes	1 year	41.1% (28/68): grade I (15), II (9), III (4)	– Mean LIF-level HU: 119.9±52.9 (grade III CS), 100.7±30.2 (grade II), 130.3±56.2 (grade I), 169.5±45 (grade 0); p < 0.01 – Segmental HU was the only independent risk factor for CS (OR 15.69, p=.017) – ROC analysis: HU cutoff of 135.02 (AUC 0.81) best predicted CS	– Revision surgery performed in 2/13 (15.4%) with severe CS and 1/55 (1.8%) without (p=.032)
Xie et al. [28]	279	TLIF *L3/4 (8), L4/5 (161), L5/S1 (110)*	Yes	NR	>2mm 29.4% (82/279)	– Mean lumbar HU: 116.1 ± 16.6 (CS group) vs. 146.0 ± 18.7 (no CS group), p<.01 – Lumbar HU, FN BMD, and lumbar BMD all independently predicted CSǁ – ROC analysis: lumbar HU (AUC 0.89) more predictive than BMD-L (AUC 0.754) and BMD-FN (AUC 0.821)	– CS associated with worse VAS leg pain (p=.02) and ODI (p=.02) at last follow-up – Nonunion rates: 14.6% (CS) vs. 8.2% (no CS), p=.07
Yao et al. [143]	93 (126 levels)	MI-TLIF *L3/4 (19), L4/ 5 (80), L5/S1 (27)*	Yes	6 wk, 3, 6, 12, and 24 mo	>2mm 34.1% (43/126), >3mm 15.9% (20)	– Mean T-scores: –1.8±1.4 (CS group) vs. –1.1±1.0 (no CS group), p=.007 – T-scores weakly correlated with amount of CS (r = –0.277, p=.006)	– CS with less ODI improvement (p=.03) and worse overall ODI (p=.04) at 2 y; no differences in VAS – Fusion at 6 mo: 83.7 (CS) vs. 78.3% (no CS), p=.47 – No revisions
Zhao et al. [144]	242	OLIF *L4/5 (242)*	Yes	1, 3, 6, and 12 mo; all cases identified within 3 mo	>2mm 32.6% (79/242), >4mm (31)	– Osteoporosis (lowest T-score from hip) an independent risk factor for CS (OR 5.976, p<.001)	– CS>4mm associated with worse ODI and VAS low back pain at 1 year (p< 0.001) – Fusion rate lower (p<.001) for CS>4mm (64.5%) vs. mild CS (83.3%) and no CS (92.6%)
Zhou et al. [29]	76 (84 levels)	OLIF *L2/3 (4), L3/4 (24), L4/5 (56)*	No	NR (minimum 6 mo)	21.2% (16/76 *), ≥2mm (7)	– Mean lumbar HU: 95.4±17.6 (CS group) vs. 136.8±28.3 (no CS group), p<.001 – Mean lowest T-scores: −2.8±0.8 (CS group) vs. −1.6±1.3 (no CS group), p<.001 – HU was the only independent risk factor for CS (UOR 0.912, p=.002) – ROC analysis: CS best predicted by HU cutoff of 115.7 (AUC 0.909) vs. lowest T-score of -2.55 (AUC 0.791)	– No difference in VAS back or leg pain or ODI at last follow-up – No recurrent radiculopathy or revisions – Fusion at last follow-up: 93.8% (CS) vs. 98.3% (no CS), p=.379

Study acronyms are explained in the first footnote to Table 3. Abbreviations: AF, anterior fixation; BPS, bilateral pedicle screw; UPS, unilateral pedicle screw; OR, odds ratio; UOR, unit odds ratio; CI, 95% confidence interval; VAS, visual analog scale; ODI, Oswestry disability index; JOA, Japanese Orthopaedic Association score; AUC, area under curve; NRS, numerical rating scale.

⁎ Given as number of patients (rather than number of cages) only.

† Unless otherwise indicated, complication rates defined as number of cages with any amount of subsidence, with severe CS referring to Grade II or Grade III CS as defined by Marchi et al.

‡ Statistical analysis performed excluding levels with lateral plate fixation.

§ Cage migration (CM): horizontal migration >2mm, cage subsidence: diagonal or vertical migration >2mm, cage retropulsion (CR): any migration into the canal or foramen.

ǁ Regression analyses performed separately with BMD-f and BMD-L as independent predictors; OR for mean HU were 1.068 (CI 1.044–1.092, p<.01) and 1.076 (CI 1.054–1.098, p<.01) in these analyses, respectively. However, only 23.7% of study participants had preoperative femoral neck DXA data and remainder obtained postoperatively, though unclear what proportion of patients ultimately had DXA data available.

Three studies did not find a significant association between BMD and CS [[31], [32], [33]]. Among these, Alan et al. [31] observed no difference in T-scores (p*=*.78) or fusion-level HU (p*=*.26) between patients with and without subsidence. However, the authors noted their study was likely underpowered given a relatively low complication rate (8 of 97 cages) and prevalence of osteoporosis (3 of 55 patients). Furthermore, all patients with low BMD were referred for preoperative endocrinology consultation with initiation of anti-osteoporosis therapy if recommended. Another potential confounder discussed was how osteoporosis status may have altered the surgical plan in favor of using supplemental pedicle screw fixation due to a presumed higher risk of subsidence. In this study, all instances of CS occurred in standalone fusions.

#### Screw loosening

**Fig. 4:**
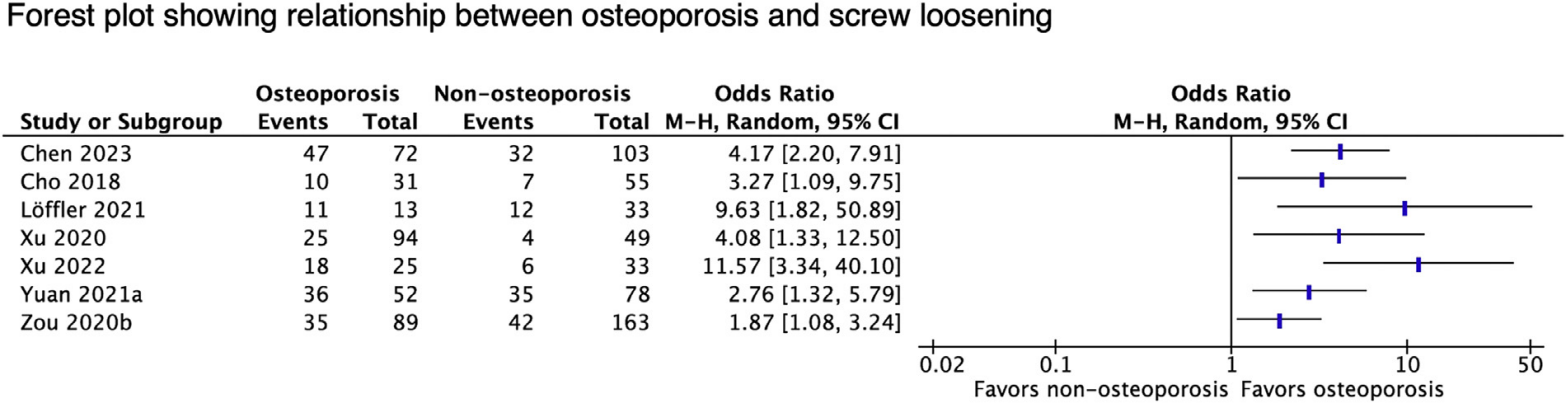
Forest plot showing relationship between osteoporosis and screw loosening.

All 14 studies reporting on screw loosening found an association between bone density and complications (Table 7). A forest plot of all contributing relevant statistics is shown in Figure 4. Three studies found higher SL rates in patients with DXA-diagnosed osteoporosis [[34], [35], [36]]. SL was also associated with HU (9 studies), vBMD (1 study), and VBQ scores (1 study). All 5 studies with comparative data found alternative metrics to be more predictive than DXA T-scores [[37], [38], [39], [40], [41]]. Zou et al. [41] demonstrated that among patients with non-osteoporotic T-scores, SL rates were significantly higher for those with HU ≤ 110 (44.4% vs. 18.6%, p<.001), suggesting that HU may be a more sensitive metric for predicting SL. Four studies measured HU along the screw trajectory, which was found to be the best predictor of loosening at those levels [39,40,42,43].

**Table 7:** Details and results of studies reporting on screw loosening.

Study	Surgical procedure *Treated levels (No.)*	Radiographic Follow-up	Complication Rates, (No. patients)	Summary of Results	Associated clinical outcomes
Bokov et al., [119]	Short-segment fusion±TLIF (162) or ALIF/D-LIF (50) *1 (153), 2 (70), 3 (21), 4 (5), 5 (1)*	6, 12, and 18 mo	38.8% (97/250)	– L3 HU was an independent risk factor for SL (UOR 0.973, p<.0001)	– 39 patients with SL had pain or disability and underwent revision.
Chen et al. [37]	Short-segment fusion with PLIF *1 (97), 2 (57), 3 (12), 4 (8)*	Median 10 mo (8-18)	29.88% (52/174), 9.18% (83/904) of screws	– Mean VBQ scores: 3.1±0.5 (SL group) vs. 2.8±0.4 (no SL group), p<.001 – Lowest DXA-measured BMD (g/cm2): 0.81±0.1 (SL group) vs. 0.86±0.1 (no SL group), p=.028 – VBQ score (per point) an independent risk factor for SL (UOR 1.02, p<.001) – ROC analysis: VBQ score 2.87 best predicted SL (AUC 0.744) – Higher rates of SL in patients with VBQ ≥ 2.9 (65.4% vs. 31.1%, p<.001)	– No patient required revision for SL
Cho et al., [34]	Single-level PLIF *L3/4 (13), L4/5 (60), L5/S1 (13)*	Mean time to SL: 6.3±3.4 mo (osteoporosis) vs. 7.3±3.0 mo (normal BMD)	19.76% (17/86)	– Mean lumbar T-scores: -2.8±0.5 (osteoporotic cohort) vs. 0.2±0.9 (normal BMD cohort), p<.001 – Higher SL rates osteoporotic cohort (32.3% vs. 12.7%, p=.029) – Mean lumbar T-scores: -1.6±1.4 (SL group) vs. -0.7±1.6 (no SL group), p=.054	– No association between SL and VAS, ODI, or EQ-5D at final follow-up – SL associated with lower fusion at 2 y (71.4% vs. 93.9%, p=.038)
Kim et al., [38]	Single or multilevel lumbosacral fusion	NR	30.97% (35/113)	– Mean L1-4 HU: 77.93±33.48 (SL group) vs. 118.79±44.59 (no SL group), p<.001 – ROC analysis: HU 104.91 (AUC 0.774) best predicted SL	N/A
Li et al., [42]	L4/5 OLIF	1 year	35.71% (40/112 levels) in 56 patients	– HU at the screw insertion site independently predicted SL at both cranial (UOR 0.971) and caudal (UOR 0.941) levels – ROC analysis: optimal HU cutoffs for predicting SL were 119.4 (AUC 0.816) for cranial and 113.75 (AUC 0.915) for caudal levels	N/A
Löffler et al. [128]	Short-segment posterior fusion *L1-5 (2), L2-5 (16), L2-S1 (8), L3-S1(18), L4-S1 (2)*	Case: 185 days (71-1359) Control: 229 days (8-2679)	Case (n=23), control (n=23)	– Mean vBMD: 86.5±29.5 (SL group) vs. 118.2±32.9 (no SL group), p=.001 – ROC analysis: optimal cutoff for predicting SL of 81.8 (AUC 0.769)	N/A
Matsukawa et al., [39]	Single-level PLIF, instrumented using CBT	1 year	13% (12/92), 4.6% (16/351) of screws	– Mean screw trajectory HU (summ): 7.68±1.8 (loosened screws) vs. 13.0±3.68 (fixed screws), p < 0.001 – Mean lumbar BMD: 0.93±0.21 (loosened screws) vs. 1.03±0.17 (fixed screws), p=.048 – Screw trajectory HU was the only independent risk factor for SL (OR 0.70, p=.018)	N/A
Sakai et al. [40]	Single-level PLIF *L1/2 (1), L2/3 (2), L3/4 (8), L4/5 (30), L5/S1 (12)*	3 mo	23% (12/52), 12% (24/206) of screws	– Mean screw trajectory HU: 147±94 (loosened screws) vs. 208±91 (fixed screws); p<.001 – Mean lumbar BMD: 1.04±0.32 (loosened screws) vs. 1.13±0.22 (fixed screws), p=.016 – ROC analysis: optimal HU cutoff of 153.5 for female (AUC 0.88) and 186.5 for males (AUC 0.635) – Screw trajectory HU was independently predictive of SL (UOR 0.989, p=.006).	N/A
Shin et al. [137]	Short-segment fusion with PLIF *1 (300), 2 (140), 3 (36), 4 (2)*	1 year	22.59% (108/478)	– Mean L4 HU: 86.9±39.4 (SL group) vs. 134.3±54.1 (no SL group), p<.01 – L4 HU was an independent risk factor for SL (UOR 0.979, p=.002) – ROC analysis: optimal HU cutoffs for predicting SL varied based on number of levels fused and postoperative C7-S1 SVA	N/A
Xu et al. [43]	L3-5 Posterolateral fusion	1 year	L3 SL 20.3% (29/143)	– Mean L3 HU (vertebral body): 98.6±25.8 (SL group) vs. 121.4±39.7 (no SL group), p<.001) – Mean L3 HU (pedicle, excluding cortical bone): 208.9±69.5 (Sl group) vs. 290.5±132 (no SL group), p=.002 – Mean L3 HU (pedicle, including cortical bone): 249.4±71.4 (SL group) vs. 337±125.5 (no SL group), p=.001 – Increased risk of L3 SL with lower L3 HU of vertebral body (OR 6.55, p=.005) and pedicle including cortical bone (OR 4.84, p=.008) – ROC analysis: SL best predicted by HU cutoffs of 130 at the vertebral body (AUC 0.674) and 340 at the pedicle (AUC 0.721)	N/A
Xu et al. [35]	Long posterior fusion to sacrum *Median 6 (range 3-12)*	NR	S1 SL 41.0% (32/78)	– Mean lumbar T-scores: −1.7 ± 1.6 (SL group) vs. −0.6 ± 2.2 (no SL group), p=.034 – Mean hip T-scores: −1.6 ± 0.7 (SL group) vs. −1.0 ± 1 (no SL group), p=.033 – Higher rates of osteoporosis in patients with SL (75% vs. 20.6%, p<.001) – Osteoporosis an independent risk factor for SL (OR 2.511, p=.002)	– Fusion rates 90.5% (SL) vs. 95.6% (no SL), p=.373 – SL did not independently predict ODI score (p=.664)
Yuan et al. [36]	Long posterior fusion *SL 6.28±1.98, no SL 5.81±1.33*	NR	54.6% (71/130), 9.4% (168/1784) of screws	– Mean T-scores: -2.12±0.96 (SL group) vs. −1.4±1.48 (no SL group), p=.002 – Higher rates of osteoporosis in patients with SL (50.7% vs. 27.12%, p<.001) – Both osteoporosis (OR 8.19, p=.001) and osteopenia (OR 5.52, p=.006) were independent risk factors for SL	– SL not associated with differences in any clinical metric
Zou et al. [145]	PLF to L5 or S1±PLIF (323) *1 (170), 2 (210), 3 (90), 4 (3)*	1 year	30.0% (151/503)	– Mean lumbar HU: 106.3±33.9 (SL group) vs. 132.6±42.9 (no SL group), p<.001 – Lumbar HU an independent risk factor for SL (UOR 0.977, p<.001) – SL rates were 4.1%, 33.3%, 53.3%, and 78.8% for 1 level, 2 levels, 3 levels, and 4 levels of fixation, respectively (OR 3.626, p < 0.001)	– Worse VAS back pain (p<.05) in SL – Higher nonunion with SL (43.0% vs. 2.6%, p < 0.001)
Zou et al., [41]	Short-segment fusion to L5 or S1±PLIF (169) *1 (78), 2 (112), 3 (45), 4 (17)*	3, 6, and 12 mo	30.6% (77/252); Most (96.1%, 172/179) at LIV or UIV	– Mean lumbar HU: 106.8±34.4 (SL group) vs. 129.8±45.7 (no SL group), p<.001 – Mean lowest T-score: -2.1±1.5 (SL group) vs. -1.7±1.6 (no SL group), p=.074 – ROC analysis: optimal HU cutoff for predicting SL of 108 (AUC 0.666) – Lumbar HU was an independent risk factor for SL (UOR 0.98, p=.002) – Higher rates of SL in osteoporotic patients (39.3% vs. 25.8%, p=.026)	– Higher nonunion at 12 mo in SL (40.3% vs. 3.4%, p<.001) – No differences in VAS or ODI

Study acronyms are explained in the first footnote to Table 3. Abbreviations: OR, odds ratio; UOR, unit odds ratio; VAS, visual analog scale; ODI, Oswestry disability index; (SRS)-22 score, scoliosis research society; AUC, area under curve.

#### Pseudarthrosis

**Fig. 5:**
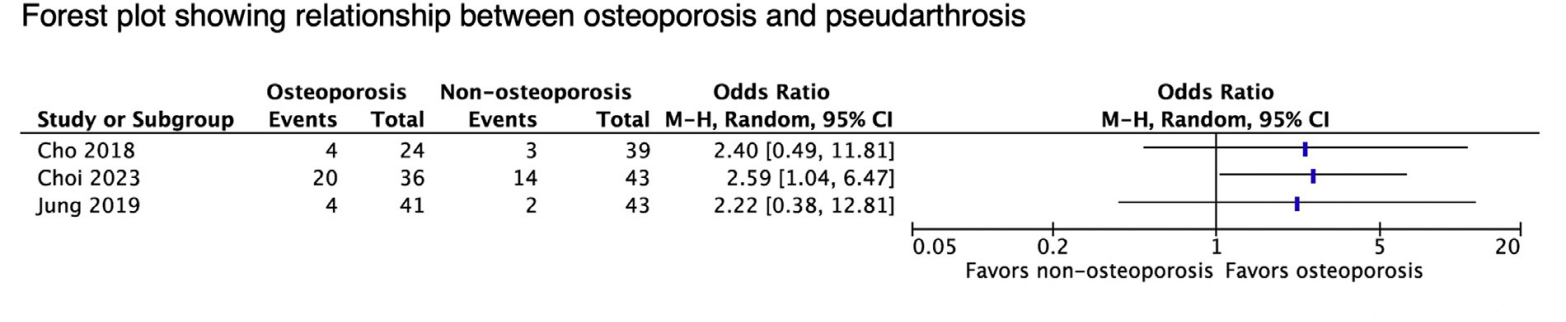
Forest plot showing relationship between osteoporosis and pseudarthrosis.

Seven studies evaluated fusion failure in relation to bone density. Criteria used to identify pseudarthrosis varied and commonly included: (1) dynamic motion at the fusion site, (2) absence of bridging trabecular bone, and 3) evidence of implant loosening (Table 8). Three studies found a significant relationship between BMD and fusion rates. A forest plot of all contributing relevant statistics is shown in Figure 5. Choi et al. [44] uniquely used 2 different CT-based radiographic criteria to investigate time to fusion after single-level TLIF and showed that osteoporosis (HU<90) was an independent predictor of slower fusion.

**Table 8:** Details and results of studies reporting on pseudarthrosis.

Study	Surgical procedure *Levels treated (No.)*	Method to assess fusion	Radiographic Follow-up	Complication Rates	Summary of Results	Associated Clinical Outcomes
Cho et al. [34]	Single-level PLIF *L3/4 (13), L4/5 (60), L5/S1 (13)*	1) segmental angulation ≤2° on dynamic x-ray, 2) absence of bridging trabecular bone or peripheral cortication on CT	1 year (XR and CT) and 2 y (XR)	6.17% (5/86)	– Mean lumbar T-scores: -2.8±0.5 (osteoporotic cohort) vs. 0.2±0.9 (normal BMD cohort), p<.001 – No difference in fusion rates at 1 year on X-ray (82.1% vs. 90.6%, p=.273) or CT (83.3% vs. 92.3%, p=.412), or at 2 y on X-ray (92.9% vs. 90.6%, p=.727)	– Fusion rates lower with SL (71.4% vs. 93.9%, p=.038), no differences based on CS (p=.4)
Choi et al. [44]	Single-level TLIF *L3/4 (7), L4/5 (47), L5/S1 (25)*	Grade 0 (nonunion): lucency visible at one or both endplates on CT Grade 1 (fusion): absence of peri-graft radiolucency Grade 2 (fusion): trabecular bone bridging	Annually to 5 y	* See footnotes	– Mean cohort times to fusion (osteoporosis vs. osteopenia vs. normal BMD) differed based on fusion criteria – Mean time to fusion for absence of peri-graft lucency: 3 y vs. 2 y vs. 0.5 y (p=.003) – Mean time to fusion for trabecular bridging: 5 y vs. 4 y vs. 3 y (p=.001) – Only L1 HU-based categorization [HU cutoffs of 90 and 120] was an independent risk factor for slow trabecular fusion (HR 0.33, p=.003)	N/A
ng , 2019 [32]	Single-level D-LIF *L1/2 (1), L2/3 (4), L3/4 (12), L4/5 (67)*	1) segmental motion (<3° or 3mm) on dynamic x-ray, 2) intervertebral bridging bone on CT, and 3) no revision or evidence of implant loosening	6 mo (XR and CT), re-evaluation at 12 and 24 mo if nonunion	5.95% (5/84)	– Mean FN T-scores: −1.7 ± 0.4 (osteopenia cohort) vs. −0.6 ± 0.6 (normal BMD cohort), p < 0.001 – No difference in cohort fusion rates at 6 mo (85.4% vs. 93.0%, p=.307), 1 year (90.2% vs. 95.3%, p=.427) or 2 y (92.7% vs. 95.3%, p=.672)	– No significant differences in VAS back or leg pain or ODI between cohorts at 1 and 2 y
Lee et al., [127]	Long posterior fusion [T10-L1 to L5/S1] with ALIF (44) or PLIF (15) *Mean 7.4±1.3*	3D-CT to assess for presence of trabecular bridging	3, 6, 9, 12, and 24 mo	L5/S1 38.98% (23/59)	– Mean T-scores: −1.31±1.81 (nonunion group) vs. −1.29±1.42 (union group), p=.799	– Patients with fusion had better ODI (p=.017) and VAS back pain (p=.035) scores at last follow-up.
Liu et al., [84]	Single-level PLIF *L4/5 (63), L5/S1 (42)*	3D-CT to assess for presence of trabecular bridging	At last follow-up (minimum 2 y)	12.38% (13/105)	– Mean BS/TV: 3.09±0.78 (nonunion group) vs. 3.71±0.76 (union group), p<.001) – Mean FN BMD (g/cm2): 0.60±0.1 (nonunion group) vs. 0.76±0.11 (union group), p=.028 – Low BS/TV was the only independent risk factor for nonunion (OR 8.53, p=.032) – ROC analysis: optimal BS/TV cutoff of 3.114 (AUC 0.807) to predict nonunion	– Higher BS/TV associated with better VAS low back and ODI at 1 and 2 y. – No differences in clinical outcome based on fusion status.
Nguyen et al. [130]	L4-S1 Posterolateral fusion	Cases identified by intractable pain with either radiographic or intraoperative evidence of nonunion	1 year	Case (n=10), control (n=10)	– Mean L4/5 HU: 166.98±23.2 (nonunion group) vs. 201.89±36.59 (union group), p=.01	N/A
Otsuki et al. [133]	L4/5 TLIF	1) Segmental dynamic motion ≤3°, 2) visible gap between cage and endplate on CT, 3) no screw loosening	1 year	26% (19/85)	– Mean fusion-level HU: 141.5±53.3 (nonunion group) vs. 141.6±44.4 (union group), p=.99	– Lower postoperative JOA (23.6 vs. 26.3) and recovery rate of JOA in nonunion (62% vs. 82%, p=.01)

Study acronyms are explained in the first footnote to Table 3. Abbreviations: OR, odds ratio; UOR, unit odds ratio; VAS, visual analog scale; ODI, Oswestry disability index; JOA, Japanese Orthopaedic Association score; AUC, area under curve; HR, hazard ratio.

⁎ At 2 years: percentage of patients demonstrating fusion with normal BMD, low BMD, and osteoporosis based on criteria of peri-graft lucency (77.1% vs. 57.2% vs. 44.6%, p=.029) and trabecular bridging (22.7% vs. 11.1%, vs. 4.0%, p=.037), respectively

#### Vertebral fracture

Six studies evaluated the relationship between osteoporosis and new vertebral fractures. Complications were identified using X-ray, CT, MRI, and/or bone scans, however, no study detailed explicit diagnostic criteria (Table 9). All 5 studies of proximal fractures found low BMD, defined by T-score [45,46] or junctional HU [[47], [48], [49]], to be an independent risk factor for new VCF. Yao et al. [49] showed not only that HU < 120 at the planned UIV strongly predicted bony PJF (OR 5.74, 95% CI 1.01-32.54, p=.04), but that there was a significant linear correlation between HU and PJK angles (r=-0.475). Due to lack of available raw data, it was not possible to create a forest plot for this outcome.

**Table 9:** Details and results of studies reporting on new vertebral fractures.

Study	Surgical procedure *Levels treated (No.)*	Radiographic Follow-up	Complication rates	Fracture location level (no.)	Summary of results	Associated clinical outcomes
Ha et al. [45]	Long posterior fusion to L5 or S1 *UIV fracture 6.2±0.4, UIV+1 fracture 5.8±1.6, no PJF 6.6±1.5*	Mean time to fracture: UIV 1.5 mo (1-4.5), UIV+1 36 mo (11-88)	7.0% (11/157) bony PJF	UIV (5), UIV+1 (6)	– Mean lowest T-scores: -3.6±0.6 (UIV fracture group), -3.6±0.8 (UIV+1 fracture group), −1.9±1.5 (no fracture group), p<.001 – Lowest T-score an independent risk factor for fracture at the UIV (HR 0.33, p=.074) and UIV+1 (HR 0.46, p=.047)	– Revision was performed in 2/5 patients with UIV fracture and none of the 6 with UIV+1 fracture
Kurra et al. [47]	Long posterior fusion to pelvis *Mean 10.7 (5-17)*	NR	11.95% (11/92) new VCF without PJK	UIV-1, UIV, or UIV+1	– New VCF patients had lower HU (sagittal, axial) at the UIV-1 (p=.05 and p=.19), UIV (p=.04 and p=.03), and UIV+1 (p=.007 and p=.02)	N/A
Luo et al. [46]	Short-segment fusion with PLIF	1, 3, 6, and 12 mo, annually thereafter	3.86% (27/669) adjacent VCF	T12 (8), T12 + L1 (1), L1 (8), L2 (5), L3 (1), L4 (2), L5 (2)	– Higher rates of osteoporosis in patients with new VCF (63% vs. 14%, p=.016) – Osteoporosis (lumbar T-score <-2.5) an independent risk factor for new VCF (OR 7.84, p=.016)	N/A
Meredith et al. [48]	Long posterior or AP fusion *Posterior (range 2-16 levels): mean fracture 6.6, control 7.3* *Anterior (range 0-7 levels): mean fracture 3.1, control 3*	Mean time to fracture 14.2 wk (2.3–45.1)	Adjacent VCF (n=20), control (n=20)	All proximal, no distal	– Mean global HU (thoracolumbar): 139.9 (new VCF group) vs. 170.1 (no fracture group), p=.032 – Mean fracture-level HU: 145.6 (new VCF group) vs. 199.4 (no fracture group), p=.006	N/A
Salzmann et al. [136]	Long posterior fusion to S1 *Fracture 5.6±3.0, control 5.1±2.4*	Mean time to fracture 87 days; 76% within 3 mo	Sacral VCF (n=21), control (n=42)	All sacral	– Mean vBMD (L1/2): 109.9 ± 35.7 (sacral fracture group) vs. 116.4±26.6 (no fracture group), p=.414 – No significant differences in experimental vBMD measurements at the S1 body (p=.567) or sacral ala (p=.616) – Obesity (OR 5.99, p=.03) was the only significant risk factor for sacral fracture	– Cases typically presented with low back or buttock pain
Yao et al. [49]	Long posterior fusion *Bony PJK 9.7±4.3, no PJK 10.75±3.9*	6 wk, 6 mo, 1 year	11.11% (7/63) bony PJK		– Mean UIV/UIV+1 HU: 109.0±22.4 (bony PJK group) vs. 168.7±66.8 (no PJK group), p=.038 – HU<120 at the UIV-UIV+1 was the only independent risk factor for bony PJK (OR 5.74, p=.04)	N/A

Study acronyms are explained in the first footnote to Table 3. Abbreviations: OR, odds ratio; HR, hazard ratio; PJA, proximal junctional angle.

#### Adjacent segment disease

**Fig. 6:**
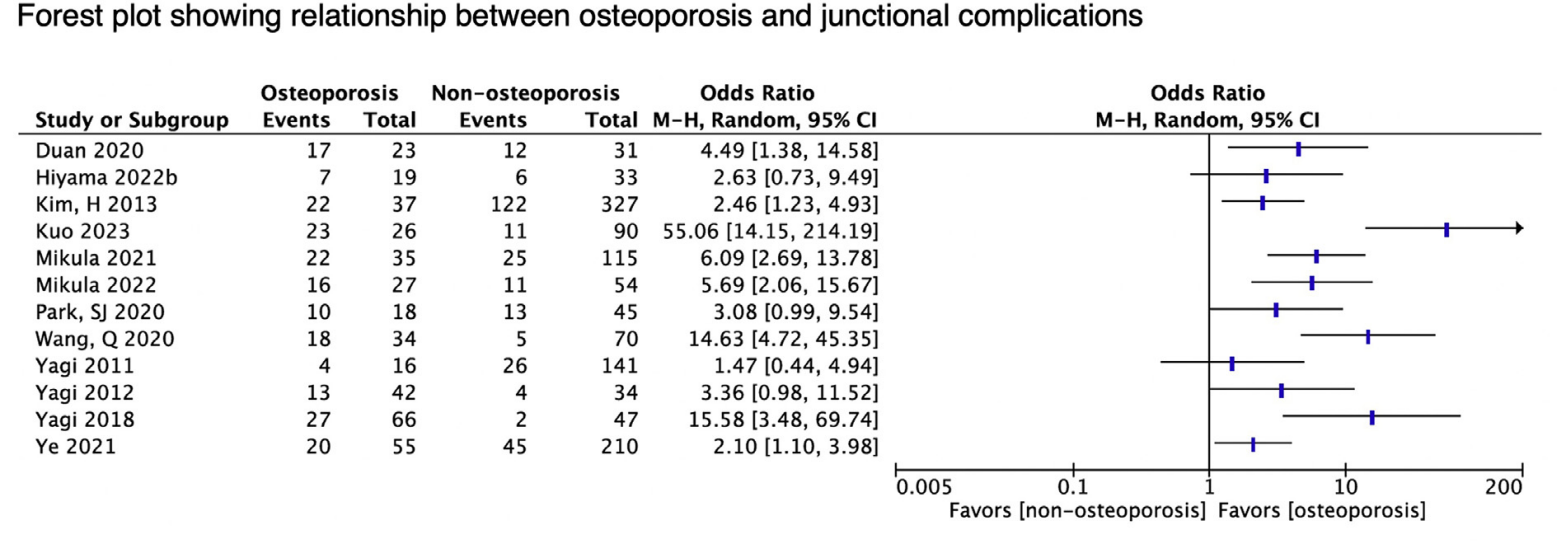
Forest plot showing relationship between osteoporosis and junctional complications.

23 studies reported on adjacent-segment complications (Table 10). A forest plot of all providing relevant data is shown in Figure 6. Most (20 studies) discussed outcomes of PJK [50] or PJF after long-segment deformity correction. Index procedures varied in terms of primary versus revision, number of fused levels, location of end-instrumented vertebrae, osteotomy, interbody fusion, and fusion to the pelvis. Studies inconsistently commented on the use of cement augmentation, proximal hooks, or other modifications to improve fixation. All studies of junctional kyphosis found low BMD to be a risk factor. Yuan et al. [51] showed that osteoporotic patients had a 14-fold increased risk of PJK (p*=*.028); at final follow-up, those with PJK had significantly worse back pain and disability scores. Park et al. [52] found a combination of 3 factors to highly predict PJF after multilevel fusion to the sacrum: age ≥70, osteoporosis, and PJA ≥0°. PJF developed in 55.6%, 73.3%, and 100% of patients with 1, 2, and all 3 characteristics, compared to none of patients without any risk factors. Of 11 studies using HU, 10 showed that values from junctional levels best predicted complications [45,47,49,[52], [53], [54], [55], [56], [57], [58]] Kuo et al.[59] performed opportunistic screening using MRI. They observed a strong linear correlation between postoperative change in PJK angles and VBQ scores (r=0.786), which was the only independent risk factor for PJK.

**Table 10:** Details and results of studies reporting on junctional complications.

Study	Surgical procedure *Levels treated (No.)*	Complications considered	Radiographic follow-up	Complication rates	Summary of results	Associated Clinical Outcomes
Barton et al. [118]	Posterior or A-P (36) fusion + osteotomy *Median posterior 8 (2-17), anterior 2 (1-6)*	PJF: fracture or spondylolisthesis of UIV or UIV+1	Between 24 and 60 mo	11.7% (11/94)	– Osteoporosis/osteopenia (DXA or ultrasound) an independent risk factor for PJF in 5+ level fusions (OR 10.4, p=.039)	– All PJF symptomatic and required revision (OR >19, p<.0001)
Chen et al., 2011 [60]	L4/5 PLIF	Progression of L3/4 degeneration: 1) disc height >3mm; 2) dynamic angulation >5°; 3) L3 slippage >3mm	Between 24 and 52 mo (at final follow-up)	22.01% (24/109)	– Mean lumbar T-scores: −1.23±0.23 (degeneration group) vs. −1.12±0.19 (no degeneration group), p=.08	– No significant differences in ODI or JOA based on BMD or degeneration
Duan et al. [53]	Long posterior fusion from T9-12 to sacrum	PJK*	< 1 month and at final follow-up	53.7% (29/54)	– Patients with PJK had lower HU at the UIV (120.41 vs. 152.8, p=.011), UIV+1 (124.52 vs. 155.96, p=.02), and UIV+2 (129.28 vs. 160, p=.018) – ROC analysis: optimal HU cutoffs at the UIV, UIV+1, and UIV+2 were 104 (AUC 0.710), 113 (AUC 0.679), and 110 (AUC 0.681) – Higher rates of PJK in patients with HU < 110 (73.9% vs. 38.7%, p=.014)	N/A
Ha et al. [45]	Long posterior fusion to L5 or S1 *PJF 6.1±1.1 no PJF, 6.6±1.5*	Acute PJF ^b^	Mean time to PJF 23.4±29.9 mo, median 8 mo (1–88)	11.5% (18/157)	– Presented differential risk profiles for PJF secondary to UIV fracture (n=5), UIV+1 fracture (n=6), UIV fixation failure (n=4), and junctional subluxation (n=3) – Mean lowest T-score: −3.3±1.1 (PJF group) vs. −1.9±1.5 (no PJF group), p<.001 – Lowest T-score was an independent risk factor for PJF (HR 0.64, p=.021)	– All patients with PJF had pain or deficits, 6 required revision.
Hiyama et al. [54]	Staged: 1) 2-4 level LLIF, 2) long posterior fusion with L5/S1 TLIF *Mean 9.7±2.5*	PJF: any symptomatic PJK requiring revision	1 year; mean time to revision 18.4±13.9 mo	25% (13/52)	– Mean UIV HU: 116.6±28.1 (PJF group) vs.141.8±41.8 (no PJF group), p=.049 – No significant differences in HU at the UIV+1 (p=.342) or UIV+2 (p=.787)	N/A
Hyun et al. [121]	Long posterior or AP (20) fusion with T9-L2 UIV *PJK 5.6±1.4, no PJK 5.6±1.3*	PJK	NR	38.6% (17/44)	– Mean T-scores: −2.5±1.2 (PJK group) vs −1.3±1.3 (no PJK group), p=.003 – Osteoporosis (T-score <-2.5) an independent risk factor for PJK (HR 2.73, p<.001)	– Lower SRS pain sub scores in PJK (p<.05), but no differences overall
Kim et al. [33]	Long posterior or AP (218) fusion	PJK: PJA > 10°	1-2 mo, 2 y, and at final follow-up	39.5% (144/364)	– Higher rates of osteoporosis in patients with PJK (20.4% vs. 9.8%, p=.02)	– Upper back pain highly predictive of PJK (OR 12.5; p<.01)
Kim et al. [125]	Long posterior or AP (32) fusion from T10-L2 to L5 or S1	PJK: angle change of >10° on dynamic x-rays	NR	32.65% (16/49)	– Mean T-scores: −2.30±0.85 (PJK group) vs. −1.01±0.67 (no PJK group), p=.027	N/A
Kuo et al. [59]	Thoracolumbar fusion	PJK and PJF requiring revision	NR	29.3% (34/116): PJK 24.1% (28), PJF 8.6% (10)	– Mean VBQ scores: 3.13±0.46 (PJF group) vs. 2.46±0.49 (no PJF group), p<.001 – VBQ score was the only independent risk factor for PJF (OR 1.74, p<.001) – ROC analysis: VBQ of 2.85 best predicted PJF (AUC 0.943) – VBQ score strongly correlated with PJA measurements (r = 0.786) – PJF developed in 26/29 (89.6%) with VBQ > 2.85 vs. 3/116 (2.5%) with VBQ < 2.85	NA
Kurra et al. [47]	Long fusion to pelvis *Mean 10.7 (5-17)*	PJK	NR	35.8% (33/92): PJK 23.9% (22), VCF excluding PJK (11)	– Mean UIV-1 HU: 131±40 (VCF group), 158±55 (PJK group), 159±45 (no PJK group) – Mean UIV+1 HU: 127±28 (VCF group), 152±50 (PJK group), 162±54 (no PJK group) – Mean UIV+1 HU: 126±33 (VCF group), 162±51 (PJK group), 171±50 (no PJK group) – No significant HU differences associated with PJK in the absence of VCF	N/A
Mikula et al. [55]	Long instrumented fusion from T10-L2 to pelvis	PJF: PJK requiring revision	Mean time to PJK 22±18 mo and PJF 19±18 mo	PJK/PJF 31.33% (47/150)	– Mean UIV/UIV+1 HU: 120 (PJK/PJF group) vs. 149 (no PJK group), p<.001 – Mean FN T-score: -1.5±1.0 (PJK/PJF group) vs. 1.0±1.0 (no PJK group), p<.05 – UIV/UIV+1 HU was the only independent risk factor for PJK (UOR 0.94, p=.031) – ROC analysis: optimal HU cutoff of 122 at UIV/UIV+1 for predicting PJK (AUC 0.89) – PJK rates for HU < 110, 110-160, and >160 were 63%, 27%, and 12% (p<.001)	N/A
Mikula et al. [56]	Long instrumented fusion from T1-T6 to pelvis	PJF: PJK requiring revision	Mean time to PJK 22 mo, PJF 14 mo	PJK/PJF 33% (27/81): PJK 26% (21), PJF 19% (15)	– Mean UIV/UIV+1 HU: 148±43 (PJK/PJF group) vs. 192±47 (no PJK group), p=.001 – Mean L3/4 HU: 91±26 (PJK/PJF group) vs. 146±49 (no PJK group), p<.05 – Mean FN T-score: -1.7±0.85 (PJK/PJF group) vs. -1.2±0.84 (no PJK group), p<.05 – UIV/UIV+1 HU was the only independent risk factor for PJK (UOR 0.96, p=.005) – ROC analysis: optimal HU cutoff of 159 at UIV/UIV+1 for predicting PJK (AUC 0.77)	N/A
Park et al., [52]	Long posterior (24) or AP (39) fusion from T11-L1 to sacrum	PJF: PJA >20°, UIV or UIV+1 fracture, UIV fixation failure, myelopathy, or need for proximal extension	Mean time to PJF 9.3±14.1 mo (1.2–55)	36.5% (23/63)	– Higher rates of osteoporosis (DXA) in patients with PJF (43.5% vs. 20%, p=.046) – Osteoporosis an independent risk factor for PJF (OR 4.459, p=.033)	– Worse ODI and SRS-22 in PJF at last follow-up – 6 (26.1%) revisions, 3 recommended but refused
Wang H et al., 2016 [57]	Long posterior fusion from T9-L3 to L4-S1	PJK or spontaneous adjacent VCF	NR	17.3% (17/98)	– Mean T-scores: −1.4±0.8 (PJK group) vs. −0.7±0.3 (no PJK group), p<.001 – Osteoporosis (T-score < -2.5) an independent risk factor for PJK (OR 3.27, p<.001)	N/A
Wang et al. [61]	TLIF (98) or PLIF (139) *1 (176), 2 (59)*	Symptomatic adjacent segment degeneration	NR	6.3% (15/237)	– Mean T-scores: -1±0.2 (degeneration group) vs. -1.2±-0.3 (no degeneration group), p=.413	N/A
Wang et al. [138]	Long instrumented fusion *Median levels: PJF 5 (4-8), control 7 (4-12)*	PJF: UIV or UIV+1 fracture, screw loosening or pullout at UIV	Median time to PJF 10 mo (2-45); 86.95% occurred within 2 y	22.1% (23/104)	– Mean L1 HU: 80±22.2 (PJF group) vs. 111±29.9 (no PJF group), p<.001) – ROC analysis: L1 HU cutoff of 89.25 best predicted PJF (AUC 0.799) – L1 HU ≤89.25 an independent risk factor for PJF (HR 8.98, p<.001) – Higher rates of PJF in patients with HU ≤ 89.25 (52.9% vs. 7.1%, p<.001)	N/A
Yagi et al., [140]	Anterior (14), posterior (82) or AP (61) fusion *Mean 10.7 (6-15)*	PJK	Final follow-up (mean 4.3 y); 75% occurred within 2 y	20% (32/157)	– Mean FN BMD: 0.691±0.194 (PJK group) vs. 0.787±0.182 (no PJK group), p=.16 – Low BMD associated with 22.9% increased risk of PJK (p=.055)	– No difference in SRS or ODI overall, but worse in symptomatic (n=6) PJK – 4 underwent revision
Yagi et al. [141]	Anterior (4), posterior (35), or AP (37) fusion *PJK 10.8±3.9, no PJK 11.2±3.6*	PJK	2-3 mo, 2 and 5 y, and at final follow-up; 76% occurred within 3 mo, none after 5 y	22.4% (17/76)	– Mean FN T-scores: -1.32±0.34 (PJK group) vs. -1.08±0.32 (no PJK group), p=.011 – Low BMD associated with 30.9% increased risk of PJK (p=.04).	– No significant differences in SRS or ODI in patients with PJK – 4 symptomatic, 2 underwent revision.
Yagi et al., 142]	Long thoracolumbar fusion *S-group 10.2±2.3, M-group 9.8±2.4*	PJF: PJA increase ≥20° with deterioration of 1+ SRS-Schwab sagittal modifier grade, or any PJK requiring revision	Within 2 y	25% (29/113) PJK, 19% (22) PJF	– Mean T-scores: −1.5±0.5 (S-group, propensity matched) vs. −0.6±0.6 (M-group), p<.001 – Higher incidence of PJF in S-group (T-score < -1.5) both before (40% vs. 4%; OR 14.3, p<.01) and after propensity-score matching (33% vs. 8%; OR 6.4, p<.01).	– 3 (2.8%) underwent revision
Yao et al. [49]	Long posterior fusion *Bony PJK 9.7±4.3, non-bony PJK 11.9±4.2, no PJK 10.75±3.9*	PJK	6 wk, 6 mo, 1 year; 65% and 87% occurred within 6 wk and 6 mo, respectively	36.5% (23/63)	– Mean UIV/UIV+1 HU: 141.7±32.4 (non-bony PJK group) vs. 168.7±66.8 (no PJK group), p=.622 – UIV/UIV+1 HU moderately correlated with PJA measurements (r = −0.475, p<.01)	– 2 required revision for progressive malalignment and intolerable pain
Ye et al. [62]	TLIF *1 (988), 2 (270)*	Symptomatic adjacent level disease requiring revision	Mean time to presentation 68.3±25.1 mo (20–123)	6.5% (65/1258)	– Incidence of DXA-diagnosed osteoporosis: 30.7% (symptomatic degeneration group) vs. 17.5% (no degeneration group), p=.069	– All symptomatic requiring revision, 2 required a second revision.
Yuan et al. [51]	Long posterior fusion with T9-L2 UIV *PJK 6.47±2.10, No PJK 5.87±1.27*	PJK	Within 6 wk and at final follow-up	20.24% (17/84)	– Mean T-scores: −2.36±0.79 (PJK group) vs. −1.61±0.91 (no PJK group), p=.01 – Osteoporosis (T-score < -2.5) an independent risk factor for PJK (OR 14.12, p=.028)	– Worse VAS low back (p=.015) and SRS-22 (p=.008) scores in PJK at final follow-up
Zhang et al. [58]	Posterior thoracolumbar fusion *PJK 4.3±1.7, No PJK 3.8 ± 1.3*	PJK	1, 3, 6, 12, 24, and 36 mo	32.4% (108/333)	– Mean UIV HU: 107.07±30.62 (PJK group, propensity matched) vs. 123.28±35.59 (no PJK group), p=.002 – ROC analysis: optimal cutoff for predicting PJK of 120.87 (AUC 0.646)	N/A

Study acronyms are explained in the first footnote to Table 3. Abbreviations: OR, odds ratio; UOR, unit odds ratio; VAS, visual analog scale; ODI, Oswestry disability index; SRS-22 score, Scoliosis Research Society; JOA, Japanese Orthopaedic Association score; AUC, area under curve; AP, anterior-posterior combined approach.

⁎ PJK defined as proximal junctional angle (PJA), measured as the sagittal Cobb between the inferior endplate of the UIV and superior endplate of UIV+2, that is both >10° and at least 10° greater than the preoperative measurement [50].

Three studies reported on adjacent segment degeneration after 1 to 2 level LIF [[60], [61], [62]]. Ye et al. [62] observed a higher incidence of osteoporosis among symptomatic patients requiring revision (30.7% vs. 17.5%; p*=*.069). The other 2 studies compared T-scores between those with and without complications. While Chen et al. [60] found affected patients had slightly lower T-scores (−1.23±0.23 vs. −1.12±0.19; p=.08), Wang et al. [61] reported no differences (-1±0.2 vs*.* -1.2±-0.3, p=.413). Notably, these studies were comprised of younger patients (mean ages 53.4 and 53.2, respectively) with relatively narrow BMD ranges.

#### Reoperation

**Fig. 7:**
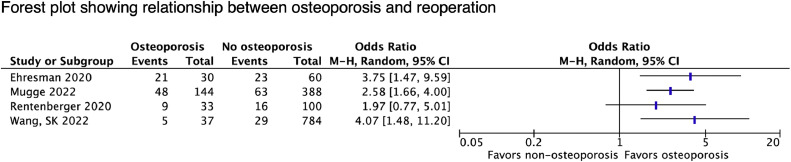
Forest plot showing relationship between osteoporosis and reoperation.

Five studies evaluated bone health as a predictor of reoperation, the timing and indications for which varied (Table 11). A forest plot of all contributing relevant data is shown in Figure 7. Wang et al. [63] showed osteoporotic patients had a 3.6-fold increased risk of reoperation within 3 months, most commonly for surgical site infection (32.3%), hematoma (23.5%), or hardware failure (20.6%). Mugge et al. [64] also observed higher revision rates with osteoporosis (33.3% vs*.* 16.2%; OR 2.93, 95% CI 1.68–5.12, p<.001), particularly for implant failures (OR 2.21, 95% CI 1.12–3.18, p=.022). Guha et al. [27] found fusion-level HU to independently predict reoperation after single or multilevel LLIF. Rentenberger et al. [65] also observed a trend towards lower vBMD in patients who required revision after SA-LLIF. In a case-control study, Ehresman et al. [66] found significantly higher VBQ scores among patients undergoing revision for symptomatic pseudarthrosis or instrumentation failure (3.29±0.68 vs. 2.92±0.46, p=.01).

**Table 11:** Details and results of studies reporting on reoperation

Study	Index surgical procedure *Levels treated (No.)*	Complications considered	Timing of reoperation	Reported rates of revision surgery	Summary of results	Associated clinical outcomes
Ehresman et al. [66]	Multilevel lumbar fusion *Case 3.6±1.1, control 3.3±0.9 (p=.106)*	Clinical or radiographic adjacent level disease or symptomatic hardware failure	Mean time to revision 3.3±2.6 y	Case (n=30), control (n=60)	– Mean VBQ scores: 3.29±0.68 (revision group) vs. 2.92±0.46 (no revision group), p=.01 – Higher rates of revision surgery in patients with VBQ >3 (70.0% vs. 38.3%, p=.005) – Mean T-score: -1.26±1.08 (revision group) vs. -0.86±0.46 (no revision group), p=.233	N/A
Guha et al. [27]	Instrumented (84) or SA-LLIF (66) *1 (54), 2+ (35)*	Revisions within 1 level of index surgery and not strictly for debridement*[]	NR	22.4% (20/89) in 28/150 levels	– Mean fusion-level HU: 131.6±50.0 (revision group) vs. 147.9±47.8 (no revision group), p=.11 – Mean FN T-scores: -1.27±1.18 (revision group) vs -1.37±0.76 (no revision group), p=.69 – Fusion-level HU an independent risk factor for revision after single- (UOR 0.98, p=.044), multilevel (UOR 0.97, p=.017), and SA (UOR 0.98, p=.02) fusions – ROC analysis: cutoff HU values for predicting revision after single and multilevel LLIF were 131.4 (AUC 0.69) and 131.0 (AUC 0.681) – Standalone surgery was a significant risk factor for reoperation (OR 186.75; p=.034)	N/A
Mugge et al., [64]	Long thoracolumbar fusion *Osteoporosis 6.7±3.6, no Osteoporosis 6.1±3.5*	Infection, neurological deficit, disease progression, construct failure (radiographic implant loosening, displacement, or fracture)	Mean time to revision 32.2±64.1 mo (osteoporosis) vs. 24.2±36.6 mo (no osteoporosis)	20.9% (111/532)	– Osteoporosis (FN T-score ≤−2.5 or history of fragility fracture) associated with increased rates of instrumentation failure (19% vs. 10%, p=.008) and need for reoperation (33% vs. 16%, p<.001) – Osteoporosis was an independent risk factor for implant failure (OR 2.21, p=.022), reoperation (OR 2.93, p<.001), and venous thromboembolism (OR 17.65, p=.03)	N/A
Rentenberger et al. [65]	SA-LLIF *1 (33), 2 (55), 3 (39), 4 (5), 5(1)*	Involving index and/or adjacent level: pain or neurologic deficit (68%), radiographic adjacent segment disease (16%), pseudarthrosis (16%), hardware failure (8%)	Within 1 year	18.79% (25/133), including 21 revised and 4 recommended	– Mean L1/2 vBMD: 96.6±35.3 (revision group) vs. 109.5±34.9 (no revision group), p=.1 – L1/2 vBMD was not independently predictive of reoperation when analyzed as either a dichotomous (p=.37) or continuous variable (OR 0.99, p=.19).	- Revision surgery not predicted by BMD or CS
Wang SK et al. [63]	Short-segment fusion with TLIF *1-2 (607), 3-5 (214)*	Early: infection (32.3%), hematoma (23.5%), implant failure (20.6%), pain (11.7%), adjacent segment disease (8.8%), CSF leak (3%) Late: adjacent segment disease (38.9%), implant failure (36.1%), infection (16.7%), pain (8.3%)	4.1% at 3 mo, 6.2% at 1 year, 8.2% at 3 y	Early (<3 mo): 4.1% (34/821) Late (>3 mo): 4.3% (36/821)	– Higher incidence of osteoporosis (T-score ≤ -2.5) in early revision group (14.7% vs. 3.9%, p=.01) – Osteoporosis was an independent risk factor for early revision (OR 3.6, p=.02)	- Worse VAS back pain at final follow-up in those who underwent revision (p=.01)

Study acronyms are explained in the first footnote to Table 3. Additional abbreviations: odds ratio (OR), unit odds ratio (UOR), visual analog scale (VAS), cerebrospinal fluid (CSF)

⁎ Indications for revision noted to be not completely recorded, but included diagnoses related to CS, pseudarthrosis, and adjacent segment disease

## Discussion

There has been a dramatic rise in the number of elective lumbar fusions performed over the past few decades, with the most significant increases occurring in patients over 65 [67]. As these procedures are associated with relatively high complication rates, particularly in elderly patients [68,69], surgeons must be aware of modifiable risk factors to allow for identification of those who may benefit from medical or surgical optimization. Biomechanically, osteoporotic bone offers poor support for instrumentation, which may predispose to failures at the implant-bone interface. In the present study, we reviewed the literature on osteoporosis as a risk factor for different mechanical complications of lumbar fusion.

An increasing number of studies have reported osteoporosis as a risk factor for cage subsidence, a finding supported by our review and others [70,71]. In osteoporotic patients, compromised vertebral strength, decreased endplate failure loads, and increased stress concentration within the surgical segment all contribute to failure at the cage-endplate interface. Surgical variables like implant design (size, shape, material properties), cage positioning on the endplate, and use of supplemental fixation are other important predictors of endplate stress and subsidence [72,73]. Selecting implants with greater endplate contact, positioning over stronger regions of the endplate, and using supplemental fixation can all help prevent subsidence [74].

Screw loosening is another common complication of lumbar fusion often associated with poor bone stock [75]. Concerns for adequate fixation in osteoporosis has prompted investigation of a number of technique modifications including cement augmentation of high-risk levels to enhance screw purchase and prevent complications [76,77]. Use of alternative screw trajectories that take advantage of stronger regions of the vertebra is also an option. BMD has a well-established association with regional vertebral strength and pedicle screw stability in vitro [78]. More specifically, BMD measurements made along a screw's trajectory can provide a particularly accurate prediction of mechanical performance and are commonly used in biomechanical investigations of fixation strength and stability [79,80]. Similar metrics are increasingly being investigated for predicting complications clinically.

Pseudarthrosis is a common consequence of implant malfunction and may be more likely in patients with osteoporosis. Among studies in this review, pseudarthrosis was most frequently reported as a secondary outcome in relation to cage subsidence or screw loosening. Park et al. [81] reported coexistence of all 3 complications: pseudarthrosis occurred in 2.9% of cages without migration compared to 45%, 58.3%, and 82.4% with migration, subsidence, and retropulsion, respectively; concomitant SL rates were 4.7%, 10%, 61.1%, and 70.6%, respectively. Unfortunately, we found relatively limited and inconclusive data regarding a direct association between bone density and fusion. One study observed that fusion took significantly longer in osteoporotic patients [44], which is consistent with findings reported by meta-analyses of randomized controlled trial data showing osteoporosis treatment improves fusion rates after lumbar instrumentation [82,83]. Liu et al. [84] published the only study in our review using micro-CT. Ex-vivo analysis of spinous process specimens obtained during index surgery revealed higher trabecular number and lower trabecular separation with greater bone surface/total volume (BS/TV) among patients ultimately achieving solid fusion. Low BS/TV was the only independent predictor of pseudarthrosis and was strongly associated with worse low back pain and disability outcomes. Although micro-CT cannot be used for preoperative risk stratification, these results can help strengthen the evidence associating bony structural deficiencies with complications.

Thoracolumbar fragility fractures are a hallmark complication of osteoporosis, usually occurring after a fall or low-energy trauma [85]. Patients who undergo long-segment fusions may be particularly susceptible to new junctional fractures under the increased stress of instrumentation. These classically occur as either (1) simple, usually chronic, compression of the first uninstrumented vertebra (UIV+1), or (2) acute UIV collapse, followed by ligamentous failure and adjacent vertebral subluxation [45,86]. The latter case is thought to be directly precipitated by significant mechanical stress from substantial alignment correction and tends to result in a more severe kyphotic deformity associated with higher rates of neurologic deficits, PJF, and revision surgery. Our review confirms osteoporosis to be a significant risk factor for PJK and PJF, particularly secondary to fracture [45,49]. In addition to considering alternative alignment targets for correction in osteoporotic patients, choosing an appropriate UIV can help minimize junctional stresses. For patients still thought to be high-risk for bony failures, prophylactic augmentation of the UIV and UIV+1 may be performed during the index procedure. There is also an abundance of evidence that perioperative anti-osteoporosis therapy can help prevent PJK. [87] Yagi et al. [88] showed that 6 months of teriparatide following thoracolumbar fusion significantly increased BMD at the UIV+1 and was associated with lower rates of bony PJK at 2 years compared to untreated controls (4.6% vs. 15.2%; p=.02).

In terms of functional outcomes, mechanical complications in this review ranged from asymptomatic radiographic findings to disabling events requiring additional surgery. In addition to construct revision for mechanical failures, several studies also found osteoporotic patients were more likely to undergo unplanned reoperations for other indications as well [64,63]. Quality of life metrics were inconsistently evaluated and represent an area of future research needed.

### Implications for practice

Collectively, our findings support the utility of osteoporosis screening prior to elective lumbar fusion. The International Society for Clinical Densitometry (ISCD) recommends preoperative bone health assessment for all females aged ≥65 years and males ≥70 years, as well as those with prior fragility fracture or other risk factors for osteoporosis [89]. However, relatively high rates of poor bone quality have been reported in younger surgical spine patients [[90], [91], [92]]. Williamson et al. [93] further demonstrated the clinical significance of this, showing that in patients under 65 with minimal deformity, osteoporosis was the most significant risk factor for major mechanical (33% vs. 7% without osteoporosis, p=.025; OR 5.9, p=.048) and major radiological (29% vs. 6%, p=.001; OR 7, p=.003) complications, trends not observed in their overall cohort. Moreover, our review suggests that patients with poor bony strength despite a non-osteoporotic BMD may also be at increased risk for mechanical complications. Thus, it may be necessary to consider alternative screening recommendations in adults presenting for elective spinal fusion [91,94,95].

Additionally, there are no current guidelines for how BMD values should be used to inform treatment in elective lumbar fusions. In particular, there has been growing interest in using site-specific HU obtained in opportunistic screening to guide risk assessment, for example using values from the UIV and adjacent levels to predict PJK. [96] However, although individual surgeons may currently employ these techniques to inform their own decision-making processes, methodologies have not been standardized and optimal thresholds for predicting complications remain unknown. Ultimately, clinical care could benefit from standardized evidence-based recommendations that reference specific BMD cutoff values and related implications for treatment.

### Limitations and future directions

The findings of this review should be interpreted in the context of its limitations. Current literature on osteoporosis and surgical outcomes is largely retrospective, which introduces a number of concerns for bias [97] Unfortunately, due to insufficient practices of osteoporosis screening, there is a relative lack of available DXA data for analysis [98,99]. As a result, many retrospective cohort studies will use International Classification of Diseases (ICD) codes to obtain information about osteoporosis status from medical records or other healthcare databases. However, it has been well-established that even among patients with documented fragility fractures, osteoporosis is profoundly underdiagnosed in both electronic medical records [[100], [101], [102]] and administrative databases [103,104]. Evaluations of osteoporosis reporting patterns in claims data have revealed the magnitude of these deficiencies, leading to recommendations against using this data in place of BMD-based reference standards [103]. In light of these limitations, the authors therefore felt it necessary to employ a more restrictive selection criteria with respect to study methodologies, focusing on comparative evaluations that explicitly investigated BMD as a predictor of mechanical complications. This excluded studies using age as a proxy for poor bone health, including those performed in elderly populations where many were likely osteoporotic but lacked attention to this diagnosis [105,106]. We also eliminated all studies in which osteoporosis status was assigned solely based on ICD code, as the absence of an osteoporosis diagnosis alone cannot be considered a reliable indicator of good bone health. Nevertheless, even among included studies, many still had potential for selection bias due to the use of imaging-based patient selection criteria, which did lower the strength of this evidence. Consideration of these limitations highlights the need for greater attention to the screening, diagnosis, and documentation of conditions like osteoporosis.

Another limitation of included studies is the potential that patients’ presumed osteoporosis status influenced the treatment they received, which could have dramatically reduced the apparent impact of osteoporosis on patient outcomes. Unfortunately, confounding variables related to the initiation, type, and duration of pharmacologic therapy as well as the use of surgical technique modifications were infrequently addressed.

Finally, studies varied considerably in patient selection criteria, surgical indications and procedures, imaging modality and anatomical site(s) of BMD assessment, and the diagnostic study, criteria, and timing used to define clinical endpoints. Together, these factors likely resulted in significant differences in both the number of complications identified and proportion attributed to osteoporosis. Use of variable and non-standardized thresholds for statistical analyses further limited any ability for direct comparison and precluded meta-analyses. Recognition of this heterogeneity is critical as it reflects the lack of consensus among surgeons and researchers regarding how to best screen for spinal osteoporosis and predict related surgical complications, concepts that are not necessarily synonymous.

The current gold-standard for assessing bony strength, skeletal fragility, and fracture risk relies on BMD, measured at the spine or hip, using DXA [25]. The anatomical site of BMD measurement is also important, as T-scores obtained from different locations are not necessarily interchangeable [107]. In a population defined by the coexistence of spinal osteoporosis and surgical degenerative pathology, measurement of BMD in the lumbar spine would theoretically be ideal for predicting focal osteoporosis-related mechanical failures [91]. However, these degenerative changes also can falsely elevate lumbar T-scores, making them paradoxically less accurate for predicting regional bony strength [108,109].

Given these shortcomings, there has been recent interest in opportunistic screening using CT or MRI, which may improve access to clinically relevant information on bone health for surgical spine patients [91,110] CT-based methods have been shown to generate reliable measures of volumetric BMD that correlate well with vertebral biomechanical properties, fracture risk, and outcomes of lumbar fusion [[111], [112], [113]]. A notable advantage of CT lies in the ability to obtain measurements from customizable regions of interest, usually located in trabecular bone and excluding osteophytes or degenerated facet joints, making them less susceptible to error from degenerative changes [114]. These measurements can also be isolated to surgically-relevant areas like pedicles, endplates, and vertebral bodies of planned instrumented levels, which may allow for personalized risk stratification and surgical decision-making. Less extensively investigated, MRI-determined VBQ scores have also shown promise for identifying osteoporosis and predicting postoperative complications [37,66,115]. As overall bony strength is determined by both bone density *and* quality, these investigations may provide important supplemental information to inform risk assessment [116].

Large-scale prospective evaluations will be necessary to evaluate the utility of these different methodologies for bone health assessment and determine which imaging study and threshold value(s) best predict mechanical complications and patient outcomes. In the meantime, it is important to consider the mounting evidence that osteoporosis is a significant risk factor for complications after lumbar fusion, and the crucial role that preoperative bone health assessment can play in mitigating these risks. Expanding access to tools like DXA, which has been widely validated and remains the current gold-standard for diagnosing osteoporosis and initiating pharmacologic therapy, will be important for identifying and treating at-risk patients [25,107,117].

## Conclusion

This systematic review provides a comprehensive summary of osteoporosis and mechanical complications of lumbar fusion. Our results demonstrate that poor bone health is an important risk factor for implant-related failure, pseudarthrosis, VCF, junctional deformity, and reoperation after elective lumbar fusion. These findings strongly support the role of preoperative screening to identify high-risk patients and allow implementation of low-risk management strategies. Our review also highlights current challenges in the evaluation and management of osteoporotic patients undergoing lumbar fusion, including a paucity of relevant and complete clinical data, variability in methods of bone health assessment and reporting of complications, and the use of heterogeneous definitions that limit the interpretation, generalizability, and meta-analysis of available evidence. As we move towards addressing these gaps, it is important to consider the mounting evidence that osteoporotic patients with degenerative spinal disease may represent a unique population in which bone health is of utmost importance, but current practices of identifying high-risk patients are inadequate. The authors therefore suggest a collaborative, multidisciplinary Academic Consortium specifically dedicated to addressing the unique challenges of treating spinal disease in patients with osteoporosis. The goals of such a consortium would begin with development of consensus criteria for best practices of bone health assessment and uniform definitions for clinical endpoint evaluation. Establishment of standardized metrics will facilitate the consistency of data collection and minimize ambiguity in a way that enables direct comparison and meta-analysis, which will be essential for adequately investigating these relationships. Ongoing integration of evolving evidence will be necessary to identify unmet needs, advance targeted research, and guide clinical decision-making towards evidence-based practice, ultimately leading to better patient outcomes.

## Declaration of competing interest

The authors declare that they have no known competing financial interests or personal relationships that could have appeared to influence the work reported in this paper.

## Appendix Supplementary materials

Image, application 1
