# Supplementary material for: The influence of osteoporosis on mechanical complications in lumbar fusion surgery: a systematic review
Source: N Am Spine Soc J. 2024 May 3;18:100327. doi: 10.1016/j.xnsj.2024.100327 (PMC11219986; doi:10.1016/j.xnsj.2024.100327)
Supplement: Supplementary file 1 [file mmc1.docx]

**Supplementary Files**

**Supplemental Table 1. Database Search Strategies and Results**

**Supplemental Table 2. Assessment of Study Quality**

**Supplemental Table 1: Database Search Strategies and Results**

| **Database** | **Search Strategy** | **Results (No. citations)** |
| --- | --- | --- |
| PubMed | ("Spine/surgery"[Mesh] OR "spine surger*" OR "spinal surger*" OR "deformity surger*" OR "reconstructive spine surgery" OR "reconstructive spinal surgery" OR "Spinal Fusion"[Mesh] OR "spinal fusion" OR "spine fusion" OR "fusion surgery" OR fusion OR "interbody fusion" OR "pedicle screw" OR "pedicle screw fixation") AND (Lumbar OR "lumbar spine" OR "Lumbar Vertebrae"[Mesh] OR "spinal deformity" OR "spine deformity" OR scoliosis OR "Scoliosis"[Mesh]) AND (osteoporosis OR "Osteoporosis"[Mesh] OR osteopenia OR "bone health" OR "Bone Density"[Mesh] OR "bone density" OR "bone quality" OR "bone mineral density" OR "bone radiodensity") AND ("complications" [Subheading] OR complications OR "Treatment Outcome"[Mesh] OR "treatment outcome" OR "outcomes" OR failure OR "mechanical failure" OR "mechanical complication" OR "clinical complication" OR "Postoperative Period"[Mesh] OR "postoperative" OR "post-operative" OR "clinical outcome" OR "radiological outcome" OR "postoperative complication" OR "Reoperation"[Mesh] OR "reoperation" OR "revision surgery") AND (adult OR "Adult"[Mesh]) AND (human OR "Humans"[mesh]) | 871 |
| Web of Science | ("spine surger*" OR "spinal surger*" OR "deformity surger*" OR "reconstructive spine surgery" OR "reconstructive spinal surgery" OR "spinal fusion" OR "spine fusion" OR "fusion surgery" OR fusion OR "interbody fusion" OR "pedicle screw" OR "pedicle screw fixation") AND (Lumbar OR "lumbar spine" OR "spinal deformity" OR "spine deformity" OR scoliosis) AND (osteoporosis OR osteopenia OR "bone health" OR "bone density" OR "bone quality" OR "bone mineral density" OR "bone radiodensity") AND (complications OR "treatment outcome" OR "outcomes" OR failure OR "mechanical failure" OR "mechanical complication" OR "clinical complication" OR "postoperative" OR "post-operative" OR "clinical outcome" OR "radiological outcome" OR "postoperative complication" OR "reoperation" OR "revision surgery") AND (adult) | 178 |
| Embase | ('spine surgery'/exp OR 'spine surgery' OR 'deformity surger*' OR 'reconstructive spine surgery' OR 'reconstructive spinal surgery' OR 'fusion surgery' OR 'fusion surgery'/exp OR fusion OR 'fusion'/exp OR 'spine fusion'/exp OR 'spine fusion' OR 'interbody fusion' OR 'interbody fusion'/exp OR 'pedicle screw' OR 'pedicle screw fixation'/exp OR 'pedicle screw fixation') AND (lumbar AND 'lumbar spine'/exp OR 'lumbar spine' OR 'spine malformation'/exp OR 'spine malformation' OR 'scoliosis'/exp OR scoliosis) AND ('osteoporosis'/exp OR osteoporosis OR 'osteopenia'/exp OR osteopenia OR 'bone health' OR 'bone health'/exp OR 'bone density'/exp OR 'bone density' OR 'bone quality'/exp OR 'bone radiodensity') AND ('outcomes'/exp OR outcomes OR 'complication'/exp OR complication OR 'failure'/exp OR failure OR 'mechanical failure' OR 'mechanical failure'/exp OR 'mechanical complication' OR 'mechanical complication'/exp OR 'clinical complication' OR 'postoperative complication'/exp OR 'postoperative complication' OR 'post operative' OR postoperative OR 'clinical outcome'/exp OR 'clinical outcome' OR 'radiological outcome'/exp OR 'radiological outcome' OR 'revision surgery'/exp OR 'revision surgery') AND ('adult'/exp OR adult) AND ('human'/exp OR human) AND [2002-2023]/py | 1103 |

**Supplemental Table 2. Assessment of Study Quality**

Quality of evidence for each study, according to the Grading of Recommendations, Assessment, Development, and Evaluations (GRADE) framework, adapted for the purposes of assessing observational studies of prognosis.

| Study | Downgrade Domains  *-1: serious risk of bias*  *-2: very serious risk of bias* | | | | | Total Downgrade | Upgrade Domains  *+1: significant effect*  *+2: very significant effect* | | Total Upgrade | Final Evidence Quality |
| --- | --- | --- | --- | --- | --- | --- | --- | --- | --- | --- |
|  | *Selection bias / Confounding* | *Inconsistency* | *Indirectness* | *Imprecision* | *Reporting / publication bias* |  | *Large effect size* | *Dose response* |  |  |
| Alan, 2022 ^25^ | 1 |  | 2 | 1 |  | **4** |  |  | **0** | Very low |
| Amorim-Barbosa, 2022 ^26^ | 1 |  |  |  |  | **1** | 1 |  | **1** | High |
| Barton, 2017 ^27^ |  |  | 1 | 1 |  | **2** | 1 |  | **1** | Moderate |
| Bokov, 2018 ^28^ | 1 |  | 1 |  |  | **2** |  | 1 | **1** | Moderate |
| Chen, 2011 ^29^ | 1 |  | 2 |  | 1 | **4** |  |  | **0** | Very low |
| Chen, 2023 ^30^ | 1 |  |  |  |  | **1** |  | 1 | **1** | High |
| Cho, 2018^31^ | 1 |  | 1 |  |  | **2** |  |  | **0** | Low |
| Choi, 2023^32^ |  |  | 1 |  | 1 | **2** | 1 |  | **1** | Moderate |
| Duan, 2020 ^33^ | 2 |  | 1 | 1 |  | **4** |  |  | **0** | Very low |
| Ehresman, 2020^34^ | 2 |  | 1 |  |  | **3** |  |  | **0** | Very low |
| Guha, 2022 ^35^ | 2 | 1 | 2 | 1 | 2 | **8** |  | 1 | **1** | Very low |
| Ha, 2019 ^36^ | 1 |  | 1 | 1 |  | **3** |  |  | **0** | Very low |
| Hiyama, 2022 a ^38^ | 2 |  | 1 |  |  | **3** |  |  | **0** | Very low |
| Hiyama, 2022 b ^37^ | 1 |  | 1 |  |  | **2** |  |  | **0** | Low |
| Hu, 2022 ^39(p)^ | 1 |  | 1 |  | 1 | **3** | 2 | 1 | **3** | High |
| Hyun, 2016 ^40^ | 1 |  | 1 |  |  | **2** | 1 |  | **1** | Moderate |
| Jones, 2021 ^41^ | 2 |  | 1 | 1 | 1 | **5** |  |  | **0** | Very low |
| Jones, 2022 ^42^ |  |  | 1 | 1 | 1 | **3** |  |  | **0** | Very low |
| Jung, 2019 ^43^ | 1 |  | 1 |  |  | **2** |  |  | **0** | Low |
| Kim HJ, 2013 ^45^ | 1 |  | 1 |  |  | **2** |  |  | **0** | Low |
| Kim MC, 2013 ^44^ | 1 |  |  |  | 1 | **2** |  |  | **0** | Low |
| Kim DK, 2017 ^46^ | 2 |  |  |  |  | **2** |  |  | **0** | Low |
| Kim KH, 2022 ^47^ | 2 |  | 1 |  |  | **3** |  |  | **0** | Very low |
| Kotheeranurak, 2021 ^48^ |  | 1 |  | 1 | 1 | **3** |  |  | **0** | Very low |
| Kuo, 2023 ^49^ | 1 |  | 1 |  |  | **2** | 1 | 1 | **2** | High |
| Kurra, 2022 ^50^ | 2 |  | 1 |  |  | **3** |  |  | **0** | Very low |
| Lee, 2020 ^51^ | 1 | 1 |  |  |  | **2** |  |  | **0** | Low |
| Li, 2023 ^52^ | 1 |  | 1 |  |  | **2** |  | 1 | **1** | High |
| Liu, 2020 ^53^ | 1 |  | 1 |  | 1 | **3** | 1 |  | **1** | Low |
| Löffler, 2021 ^54^ | 2 |  | 2 |  | 1 | **5** |  |  | **0** | Very low |
| Luo, 2020 ^55^ | 2 |  |  |  |  | **2** | 1 |  | **1** | Moderate |
| Matsukawa, 2018 ^56^ | 1 |  | 1 |  | 1 | **3** |  |  | **0** | Very low |
| Meredith, 2013 ^57^ | 2 |  | 1 |  |  | **3** |  |  | **0** | Very low |
| Mi, 2017 ^58^ | 1 |  | 2 |  |  | **3** |  |  | **0** | Very low |
| Mikula, 2021 ^59^ | 1 |  | 1 |  |  | **2** |  | 1 | **1** | Moderate |
| Mikula, 2022 ^60^ | 1 |  | 1 |  |  | **2** |  | 1 | **1** | Moderate |
| Mugge, 2022 ^61^ |  |  |  |  |  | **0** | 1 |  | **1** | High |
| Nguyen, 2015 ^62^ | 2 |  | 2 | 1 |  | **5** |  |  | **0** | Very low |
| Oh , 2015^63^ | 1 |  | 1 |  | 1 | **3** | 1 | 1 | **2** | Moderate |
| Okano, 2020 ^64^ | 2 |  | 1 |  | 1 | **4** |  |  | **0** | Very low |
| Otsuki, 2021 ^65^ | 2 |  | 2 | 1 |  | **5** |  |  | **0** | Very low |
| Park MK, 2019 ^66^ | 1 |  | 1 |  | 1 | **3** | 1 |  | **1** | Low |
| Park SJ, 2020 ^67^ | 1 |  |  |  |  | **1** | 1 |  | **1** | High |
| Pisano, 2020 ^68^ | 2 |  | 1 |  |  | **3** |  |  | **0** | Very low |
| Pu, 2022 ^69^ | 1 |  |  |  |  | **1** |  |  | **0** | Moderate |
| Ran, 2022 ^70^ | 2 |  | 1 |  |  | **3** |  |  | **0** | Very low |
| Rentenberger, 2020 ^71^ | 1 |  | 1 |  | 1 | **3** |  |  | **0** | Very low |
| Sakai, 2018 ^72^ |  |  | 1 | 1 | 1 | **3** |  | 1 | **1** | Low |
| Salzmann, 2019 ^73^ | 1 |  | 1 | 1 |  | **3** |  |  | **0** | Very low |
| Shin, 2022 ^74^ |  |  | 1 |  |  | **1** |  | 1 | **1** | High |
| Wang H, 2016 ^75^ | 1 |  | 1 | 1 |  | **3** | 1 |  | **1** | Low |
| Wang H, 2017 ^76^ | 1 |  | 2 |  |  | **3** |  |  | **0** | Very low |
| Wang Q, 2020 ^77^ | 1 |  | 1 |  |  | **2** | 1 |  | **1** | Moderate |
| Wang SK, 2022 ^78^ |  |  |  |  |  | **0** | 1 |  | **1** | High |
| Xi, 2020 ^79^ | 1 |  | 1 | 1 |  | **3** | 2 |  | **2** | Moderate |
| Xie, 2022 ^80^ | 2 |  | 1 |  | 1 | **4** |  |  | **0** | Very low |
| Xu, 2020 ^81^ | 1 |  | 1 |  |  | **2** | 1 |  | **1** | Moderate |
| Xu, 2022 ^82^ | 1 |  |  | 1 |  | **2** | 1 |  | **1** | Moderate |
| Yagi, 2011 ^83^ | 1 |  | 2 | 1 |  | **4** |  |  | **0** | Very low |
| Yagi, 2012 ^84^ | 2 |  | 2 | 1 |  | **5** | 1 |  | **1** | Very low |
| Yagi, 2018 ^85^ | 1 |  |  | 1 |  | **2** | 1 |  | **1** | Moderate |
| Yao, 2020 ^86^ | 2 |  |  |  | 1 | **3** |  | 1 | **1** | Low |
| Yao, 2021 ^87^ | 1 |  | 1 | 1 |  | **3** | 1 | 1 | **2** | Moderate |
| Ye, 2021 ^88^ | 2 |  | 1 |  |  | **3** |  |  | **0** | Very low |
| Yuan, 2021 a ^90^ |  |  |  |  |  | **0** | 2 |  | **2** | High |
| Yuan, 2021 b ^89^ |  |  |  |  |  | **0** | 1 |  | **1** | High |
| Zhang, 2022 ^91^ | 1 |  | 1 |  |  | **2** |  |  | **0** | Low |
| Zhao, 2022 ^92^ | 1 |  |  |  | 1 | **2** | 1 |  | **1** | Moderate |
| Zhou, 2021 ^93^ |  |  |  |  | 1 | **1** |  | 1 | **1** | High |
| Zou, 2020 a ^94^ | 1 |  | 1 |  |  | **2** |  | 1 | **1** | Moderate |
| Zou, 2020 b ^95^ | 2 |  |  |  |  | **2** |  | 1 | **1** | Moderate |
